# Biofouling-Resistant Ultrafiltration Membranes via Codeposition of Dopamine and Cetyltrimethylammonium Bromide with Retained Size Selectivity and Water Flux

**DOI:** 10.1021/acsami.2c05844

**Published:** 2022-08-10

**Authors:** Aydın Cihanoğlu, Jessica D. Schiffman, Sacide Alsoy Altinkaya

**Affiliations:** † Faculty of Engineering, Department of Chemical Engineering, İzmir Institute of Technology, 35430 Urla-İzmir, Turkey; ‡ Department of Chemical Engineering, University of Massachusetts Amherst, Amherst, Massachusetts 01003-9303, United States

**Keywords:** anti-biofouling, biofouling resistance, polydopamine, ultrafiltration membrane, quaternary ammonium compounds

## Abstract

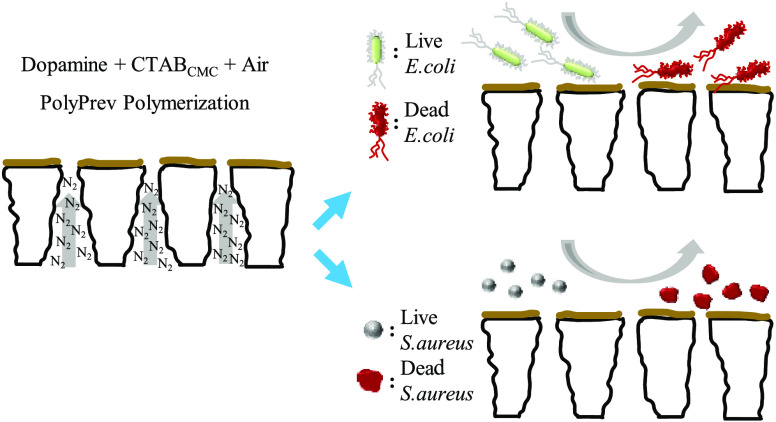

Biofouling is a serious problem in ultrafiltration (UF) membrane applications. Modifying the surface of membranes with low molecular weight, commercially available antibacterial chemistries is an excellent strategy to mitigate biofouling. Herein, we report a new strategy to impart antibacterial and anti-biofouling behavior without changing the support membrane’s size selectivity and pure water permeance (PWP). To this end, a strong antibacterial agent, cetyltrimethylammonium bromide (CTAB), was codeposited with dopamine onto commercial polyethersulfone (PES) UF membranes in the presence of nitrogen (N_2_) gas backflow. The PWP and pore size of the support membrane did not change with codeposition, confirming the benefit of N_2_ backflow in mitigating the solution intrusion phenomenon. X-ray photoelectron spectroscopy (XPS), surface ζ potentials, and contact angle measurements confirmed the successful codeposition of polydopamine (PDA) and CTAB onto the membrane. Among three different CTAB concentrations systematically investigated, the membrane functionalized with CTAB at the critical micelle concentration (CMC) provided the best anti-biofouling activity against Gram-positive (*Staphylococcus aureus*) and Gram-negative (*Escherichia coli*) bacteria and retained its surface ζ potential after being stored in 1 M NaCl (pH = 6.8) for 3 months. Our results demonstrate the potential of using a facile, one-step approach to modify commercial UF membranes without compromising their pore size or flux, while simultaneously endowing antibacterial activity.

## 1 Introduction

Biofouling is detrimental in membrane-based technologies and is caused by the attachment of living microorganisms, such as bacteria and algae, to the surface of membranes. Unlike other foulants, living microorganisms proliferate and quickly form a cohesive biofilm on the surface of the membrane.^1^ To continue to produce clean water after fouling occurs requires higher energy consumption and increased maintenance costs because the membranes have to be cleaned using harsh chemicals or completely replaced. Ultrafiltration (UF) and microfiltration (MF) membranes are widely used in industrial applications to effectively remove bacteria and algae. The membranes are comprised of polysulfone (PSF), polyethersulfone (PES), and poly(vinylidene fluoride) (PVDF).^2,3^ Unfortunately, these membranes have a high propensity to biofouling due to their lack of antibacterial and/or hydrophilic chemical functionality. Membrane surface modification is considered a feasible strategy to increase the fouling resistance of commercial membranes.^4^

Several research groups have used antimicrobial polymers,^5^ quaternary ammonium compounds (QAC),^6^ and zwitterionic polymers^7^ to create antibacterial and/or antifouling surfaces via surface-initiating polymerization,^8^ grafting,^9^ mussel-inspired chemistry,^10^ and layer-by-layer assembly.^11^ Among the techniques mentioned, mussel-inspired polydopamine (PDA) has attracted great interest in membrane modification over the last decades due to its material-independent surface functionalizing capability and presence of catechol and amine groups, which simultaneously make the surface hydrophilic and also enable further chemical modifications.^12^ For example, while membranes coated with a pure PDA layer showed an increased initial fouling resistance against organic foulants, such as oil emulsions^13^ and bovine serum albumin (BSA),^14^ limited antibacterial activity against Gram-positive and Gram-negative bacteria was demonstrated.^15,16^ Therefore, researchers have used a one-step process to produce biofouling-resistant membranes using the bioinspired “glue” PDA to codeposit additional functional molecules, such as zwitterions^15−19^ and reduced graphene oxide–copper nanocomposites.^20^ Although the prepared membranes demonstrated enhanced hydrophilicity and good antibacterial activity, a significant flux decline was observed due to the penetration of monomers, polyelectrolytes, and polymers into the porous support.^15−17^ To mitigate this solution “intrusion phenomenon”, an interlayer of cellulose nanocrystals (CNCs),^21^ carbon nanotubes,^22^ PDA-wrapped carbon nanotubes,^23^ or cadmium hydroxide (Cd(OH)_2_) nanowires^24^ have been applied onto porous MF and UF supports before interfacial polymerization. However, both the Cd(OH)_2_ nanowires and the carbon nanotubes are toxic to the environment. Although the CNC is nontoxic, its production is not environmentally friendly due to the highly concentrated acid solution needed for the hydrolysis of cellulose.

Recently, Dobosz et al. developed a method that modified only the surface of UF membranes while avoiding modifying the pores.^25^ The method, abbreviated as PolyPrev (the polymer prevention system), creates an inert physical barrier within the pores of the membrane by backfilling the pores from the bottom (support side) of the membrane with inert N_2_ gas. This strategy enabled pure PDA-modified UF membranes to retain the same flux and pore size as the unmodified membranes. After a 24 h incubation period, the membranes functionalized with PDA alone had a high coverage by *Escherichia coli* (83 ± 12%) relative to the unmodified (control) membrane. When the same system was used to codeposit PDA with a polymer zwitterion or end-functionalized poly(ethylene glycol), there was a significant reduction in the number of *E. coli* cells that attached to the surface. Although such antiadhesive hydrophilic surfaces are effective for controlling the initial adsorption of bacteria, they cannot prevent the growth and multiplication of microorganisms or inactivate the irreversibly adhered microorganisms. Thus, new surface modifications are needed that significantly advance the antibacterial properties of the membranes without altering their permeabilities.

The current study proposes a one-step process that modifies the surface of commercial UF PES membranes by simultaneously codepositing the surfactant cetyltrimethylammonium bromide (CTAB) with dopamine. CTAB was selected because it is a low-cost additive that has a low molecular weight and high commercial availability.^26−29^ Its low persistence, bioaccumulation, and mobility in water and soil^30^ are other advantages of using CTAB for surface modification. Additionally, CTAB has a strong antibacterial activity against both Gram-positive and Gram-negative bacteria due to its ideal chain length of 16.^31^ Previous studies have revealed that the antibacterial activity of QACs increased when the carbon chain length was increased from 3 to 16, but then decreased at greater chain lengths, i.e., 18.^32^ To date, QACs that have been integrated into the body or on the surface of membranes either had a short chain length^33−35^ or a high chain length (>16),^36^ but none of these studies explored the antibacterial activity of the CTAB-containing membranes. The studies reported improved membrane flux, hydrophilicity, and solute retention characteristics upon CTAB addition.^37−39^ Only recently, we have demonstrated that by adding CTAB into the coagulation bath used during fabrication that membranes are rendered antibacterial.^40^ A high CTAB concentration at the surface of the membranes was achieved due to its electrostatic interaction with the sulfonated polyethersulfone (SPES) at the polymer/bath interface. However, this one-step protocol was only successful if the polymer membrane offered functional groups, such as hydroxyl, carboxyl, amine, and sulfonic. Unfortunately, the polymers commonly used in the manufacture of commercial membranes lack such functional groups. Thus, a universal and facile surface modification that deposits CTAB on the surface of any polymer membrane holds promise but has not yet been demonstrated. Our approach proposed in this work has three essential advantages. First, CTAB and dopamine cannot penetrate into the pores of the membrane because the PolyPrev continuously plugs them with the inert gas. Thus, flux reduction due to pore narrowing is minimized, while exposure to the antibacterial agent is maximized. Second, because CTAB has a low molecular weight, a thin antibacterial coating that does not alter the membrane’s flux is formed. Third, a stable complex is formed because the positively charged CTAB electrostatically interacts with the negatively charged dopamine as it polymerizes into PDA. Here, we systematically explored the CTAB concentration above, at, and below the critical micelle concentration to examine its effect on membrane properties, including its biofouling resistance to Gram-positive (*Staphylococcus aureus*) and Gram-negative (*E. coli*) bacteria using dynamic filtration experiments. To the best of our knowledge, this is the first study that developed an antibacterial and biofouling-resistant UF PES membrane through the effective deposition of a low-molecular-weight surfactant.

## 2 Experimental Section

### 2.1 Materials

Dopamine hydrochloride (Scheme S1a), tris hydrochloride buffer, and sodium hydroxide were purchased from Sigma-Aldrich. Cetyltrimethylammonium bromide (CTAB, MW: 365 Da) (Scheme S1b) was supplied by Alfa Aesar and used as an antibacterial agent. Phosphate-buffered saline (PBS), sodium chloride (NaCl), and isopropyl alcohol (IPA) were obtained from Sigma-Aldrich. Sodium hydroxide (NaOH) and hydrochloric acid (HCl) with 37% purity used for pH adjustments were purchased from Sigma-Aldrich and Merck, respectively. Gram-negative (*E. coli*, ATCC 25922) and Gram-positive (*S. aureus*, RSKK 1009) bacteria were used in anti-biofouling tests. The commercial PES UF support membranes (NADIR PM UP150) with a reported nominal molecular-weight limit of 150 kDa were supplied by MicroDyn Nadir. Deionized water with a conductivity of 0.05 μS/cm was used for the experiments. All chemicals were used as received without further purification.

### 2.2 Modification of Membranes with Polydopamine

**Scheme 1:**
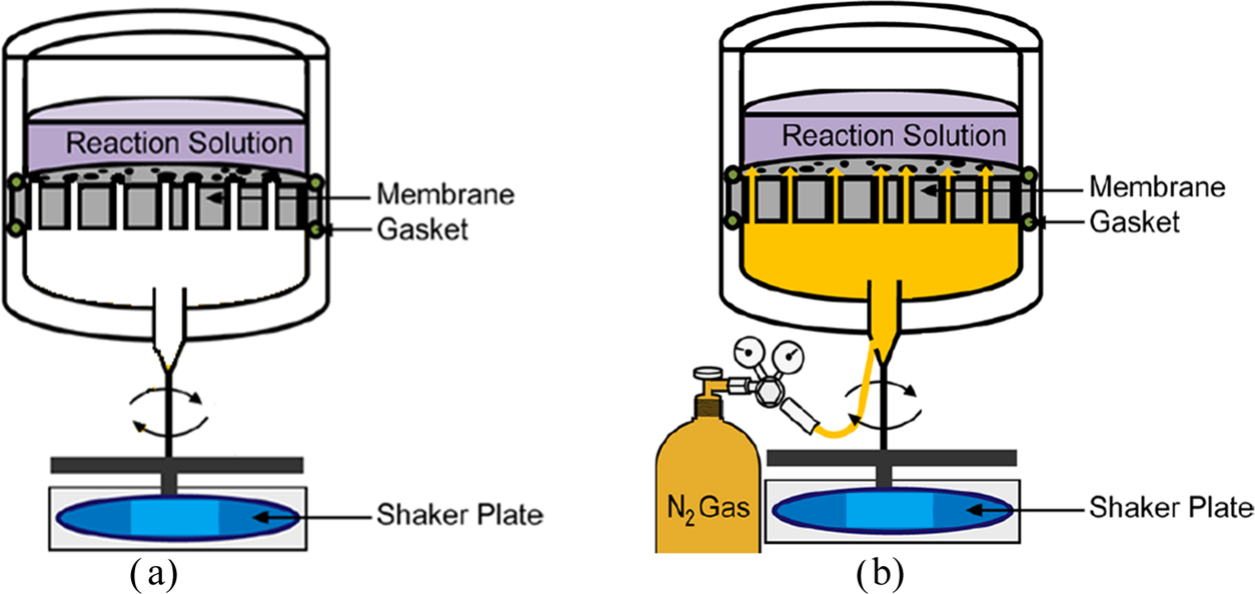
(a) Conventional Polymerization System and (b) PolyPrev Polymerization System (Reprinted with Permission from Reference (25), Copyright (2019) American Chemical Society)

Before using the commercial membrane coupons as supports, they were pretreated by immersion into 25% (v/v) IPA solution for 1 h, followed by overnight storage in deionized water. The pretreated membrane coupons were placed in a custom-designed coating device (Scheme 1) that limited the coating to only one side (active side) of the membrane. Next, the reaction solution (50 mL), consisting of dopamine hydrochloride (2 mg/mL) dissolved in Tris-HCl buffer solution (10 mM, pH 8.5) at room temperature (25 °C), was poured onto the active side of the membrane and stirred gently at 100 rpm. In the conventional polymerization system, there was no nitrogen (N_2_) backflow (Scheme 1a). In contrast, in the PolyPrev polymerization system, N_2_ was continuously fed at 0.3 bar during the polymerization to prevent the diffusion of the molecules into the pores (Scheme 1b). The polymerization was carried out at room temperature (25 °C) for 1 h. The membranes modified by the conventional and PolyPrev polymerization systems are labeled PES_PDA_Conv Poly_ and PES_PDA_PolyPrev Poly_, respectively, throughout the Results Section 3.

### 2.3 Modification of Membranes with Codeposition

The codeposition reaction solution was prepared by dissolving CTAB in dopamine solution at room temperature (25 °C). Three different CTAB concentrations were explored, including 10^–4^ M (lower than the critical micelle concentration (CTAB_LCMC_)), 10^–3^ M (the critical micelle concentration (CTAB_CMC_)), and 10^–2^ M (higher than the critical micelle concentration (CTAB_HCMC_)); the resulting membranes will be referred to as PES_PDA + CTAB_LCMC_, PES_PDA + CTAB_CMC_, and PES_PDA + CTAB_HCMC_, respectively, throughout the results. The support membranes underwent the same pretreatment protocol, as explained in Section 2.2. All membranes were codeposited by applying a 100 rpm shaking rate at room temperature (25 °C) for 1 h in the presence of N_2_ backflow (0.3 bar). Scheme S1 details the membrane fabrication steps.

### 2.4 Membrane Filtration Performance

Pure water permeance (PWP) of the support and modified membranes was determined using a 200 mL dead-end stirred cell (Millipore, Amicon Stirred Cell UFSC20001) with an effective area of 28.7 cm^2^. Before any filtration test, first, membrane coupons were compacted at 1 bar using pure water until the flux was stable. Next, pure water was filtered at 0.5 bar and the collected permeate volume was recorded at specific time intervals. The volumetric flux was calculated from the slope of the permeate volume vs. time graph and converted to hydraulic PWP using the following equation
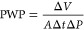
1where Δ*V* is the volume of permeated water (L), *A* (m^2^) is the membrane area, Δ*t* (*h*) is the permeation time, and Δ*P* (bar) is the transmembrane pressure difference applied through the membrane (*n* = 3), where “*n*” represents the repeat number of experiments.

### 2.5 Membrane Characterization

The structure and elemental compositions of the membranes were determined using X-ray photoelectron spectra (XPS) (Thermo Scientific) at the emission angles of 0° (*n* = 3). The surface ζ potential measurement (NanoPlus Micromeritics Instrument) of the membranes (16 mm × 37 mm) was carried out with the 10^–2^ M NaCl electrolyte solution (*n* = 3) using a quartz glass flat surface cell. The pH of the electrolyte solution was adjusted using HCl and NaOH. The surface hydrophilicity of the membranes was determined using contact angle measurements (Attension optical tensiometer) using a 5 μL deionized water droplet (*n* = 5). The surface morphology of the membranes was visualized using a scanning electron microscope (SEM, FEI Quanta 250 FEG). Gold was coated on the membrane surface with a Magnetron Sputter Coating Instrument before taking SEM micrographs. The average pore diameter distributions were determined by measuring at least 30 random pores present on the high-magnification SEM images using *ImageJ* software (National Institutes of Health, Bethesda, MD).^25^ The surface roughness of the membranes (arithmetic mean (Ra) and root-mean-square (Rq)) was determined using an atomic force microscope (AFM) (MMSPM Nanoscope 8, Bruker). A sample area (5 × 5 μm) was scanned at a rate of 1 Hz using tapping mode in the air at room temperature using a TAP150 model tip (Bruker) (*n* = 3). Before XPS, SEM, AFM, and contact angle measurements, all membrane coupons were dried in a vacuum oven overnight at room temperature (25 °C).

### 2.6 Coating Thickness Measurements

Clean n-type crystalline silicon wafers (c-Si) (University Wafers, MA) were immersed in the pure PDA and codeposition solution for 24 and 72 h, respectively, and during coating, the shaking rate was adjusted to 100 rpm (*n* = 3). Following overnight drying of coated silicon wafers at 25 °C in an oven, the thicknesses of the pure PDA and codeposited layers were measured using a reflectometer system (MProbe-Vis20) with a spectral range of 400–1100 nm.

### 2.7 Analysis of the Anti-Biofouling Performance

The biofouling of the support and modified membranes was determined using dynamic filtration experiments using the model microbes *E. coli* and *S. aureus*, as previously reported.^40^ A dead-end cell filtration system with a cell volume of 50 mL and an effective surface area of 13.4 cm^2^ (Millipore, Amicon Stirred Cell 8050) was employed. Before bacterial filtration, each side of the membrane coupons was sterilized using UV light for 10 min. *E. coli* and *S. aureus* bacterial suspensions were prepared in PBS (pH 7.4) to reach concentrations of 1.8 × 10^8^ and 2.1 × 10^8^ CFU/mL, respectively. Bacterial suspensions (250 mL) were filtered through the support and modified membranes where the initial fluxes of the membranes were adjusted to the same values. Following filtration, the membrane coupons were rinsed with PBS for 10 min and the water flux was remeasured to calculate the flux recovery ratio (FRR).
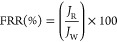
2where *J*_W_ is the pure water flux of the clean membrane and *J*_R_ is the pure water flux of the washed membrane. The experiments were carried out at room temperature (25 °C).

### 2.8 Analysis of the Antibacterial Performance

The antibacterial activity of the membranes was performed using the colony-counting method according to the American Society for Testing and Materials (ASTM-E2180) standard protocol.^40^ Briefly, the final concentrations of Gram-positive (*S. aureus*) and Gram-negative (*E. coli*) bacterial suspensions were adjusted to 3.5 × 10^6^ and 4.2 × 10^6^ CFU/mL, respectively. Each side of the membrane coupons (effective area 3 cm × 3 cm), consistent with the ASTM standard recommendation, was first sterilized using UV light for 15 min before being placed on agar plates. Next, 300 μL of the bacterial suspension was poured onto the active side of the membranes and incubated for 24 h at 37 °C. Following incubation, the membranes were put into Erlenmeyer flasks containing 50 mL of phosphate-buffered saline solution (PBS, pH = 7.4), and subjected to sonication for 10 min to remove the deposited bacteria from the membrane surface. Finally, the bacterial suspensions were spread on LB plates, incubated for 24 h at 37 °C, and the colonies were counted. The bactericidal rate was calculated using the following equation
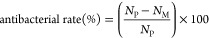
3where *N*_P_ and *N*_M_ are the numbers of visual bacterial colonies on the agar plates after contact with the support and modified membranes, respectively (*n* = 3).

### 2.9 Chemical Stability of the CTAB Coating

Stability experiments were performed on the CTAB coating (PES_PDA + CTAB_CMC_) that demonstrated the highest biofouling resistance. The coated membrane was stored in 1 M NaCl solution for 3 months under static conditions (no shaking) at 25 °C. The surface ζ potential of the stored membrane was compared to that of the fresh membrane (*n* = 3).

The alkaline stability of the coating layer on the PES_PDA_PolyPrev Poly_ and PES_PDA + CTAB_CMC_ membranes was tested using 0.1 M NaOH solution (pH = 13). To this end, the membrane coupons (28.7 cm^2^) were immersed in 50 mL of alkaline solution for 6 and 24 h under static conditions at 25 °C. Then, the released PDA in solution was determined with UV–vis measurement at a 420 nm wavelength.

## 3 Results and Discussion

### 3.1 Performance and Characteristics of PDA-Coated Membranes

**Figure 1:**
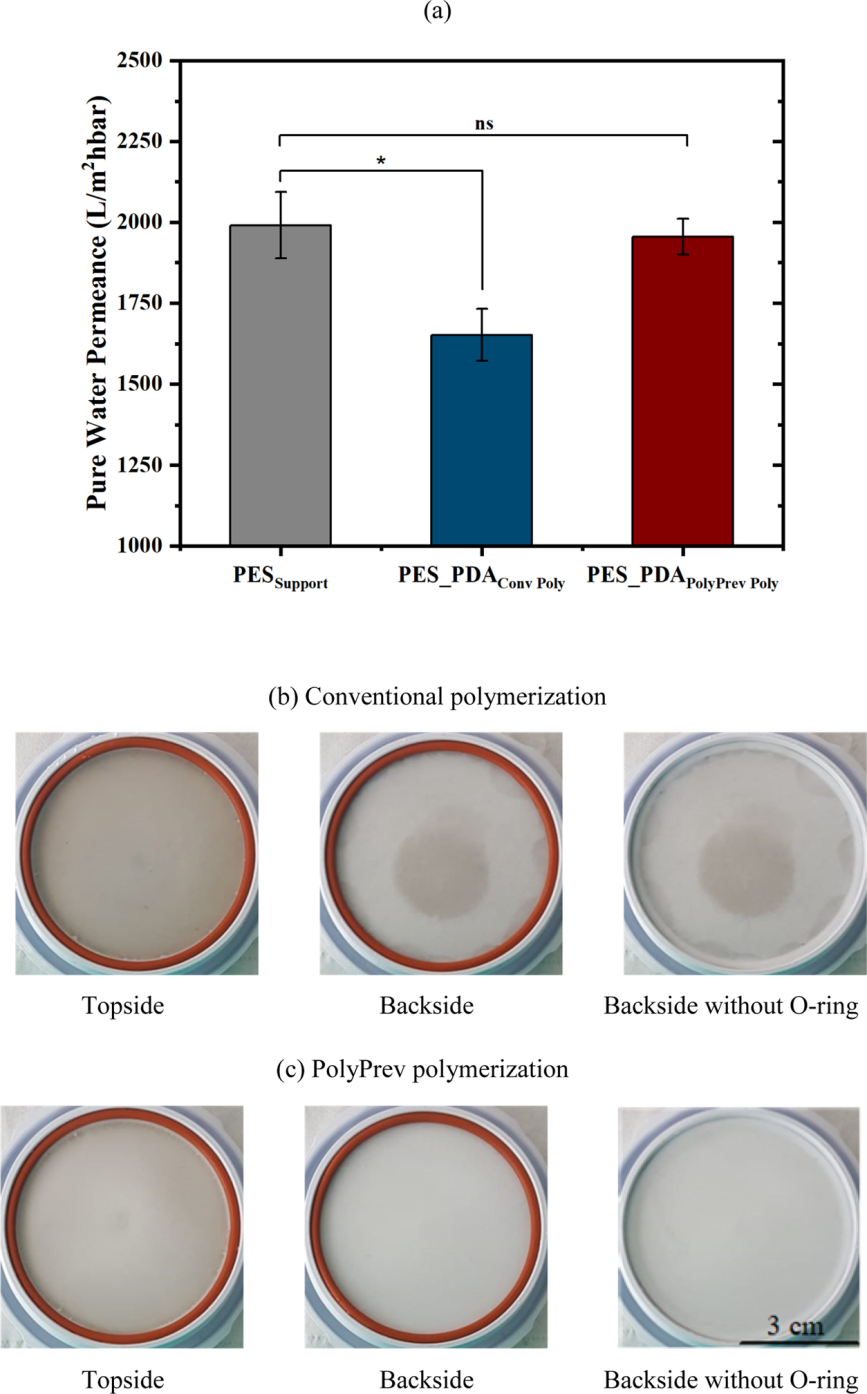
(a) Pure water permeance of the membranes. *Represents a statistically significant difference (*p* < 0.05) in pure water permeance of the PES_Support_ and PES_PDA_Conv Poly_ membranes. ns represents a statistically insignificant difference (*p* > 0.05) in pure water permeance of the PES_Support_ and PES_PDA_PolyPrev Poly_ membranes. Digital images of the top and backside of the (b) PES_PDA_Conv Poly_ and (c) PES_PDA_PolyPrev Poly_ membranes.

In this work, a commercial PES UF membrane with a trade name of PM UP150 (Germany) was chosen as the support because it is commonly used for membrane bioreactor (MBR) applications. Thus, improving the biofouling resistance, while maintaining consistent water permeance using these PES membranes is critical for prolonging their lifetime and reducing treatment costs. While conducting conventional dopamine polymerization on the surface of porous supports seemed like a promising approach, the literature has demonstrated that over time, membranes functionalized with PDA alone experience a severe flux reduction due to the solution intrusion phenomenon, which causes two limitations (1) pore narrowing and (2) fouling.^41^ In this work, we too found that the PES membrane modified using the conventional polymerization technique had a statistically lower flux than the unmodified membrane (Figure 1a).

To address the first limitation of conventional dopamine-only polymerization, i.e., the transport of dopamine into the pores, we created an inert physical barrier inside the pores using a continuous backflow of N_2_ during polymerization (Scheme 1).^25^Figure 1b,c shows digital images of the dense side of the membrane (topside), which was in contact with the dopamine solution, and the porous side (backside) of the support. The bottom of the membrane modified with N_2_ backflow remained white (Figure 1c), while in the absence of gas flow (conventional polymerization), it was a characteristic brown color (Figure 1b), which suggests that the polymerization also took place inside the pores. Notably, the images of the PES_PDA_PolyPrev Poly_ membrane had a less intense brown color, which could be explained by the reduced dissolved oxygen concentration in the presence of N_2_ backflow where the dissolved oxygen and alkaline environment are essential requirements for dopamine polymerization.^25^ Next, we explored the pure water permeance of the membranes. The polydopamine coating formed with N_2_ flow had a statistically equivalent permeance as the PES_Support_ (Figure 1a). Both the visual images and permeance results support the fact that the N_2_ gas flow prevented the penetration of the monomer into the pores.

**Figure 2:**
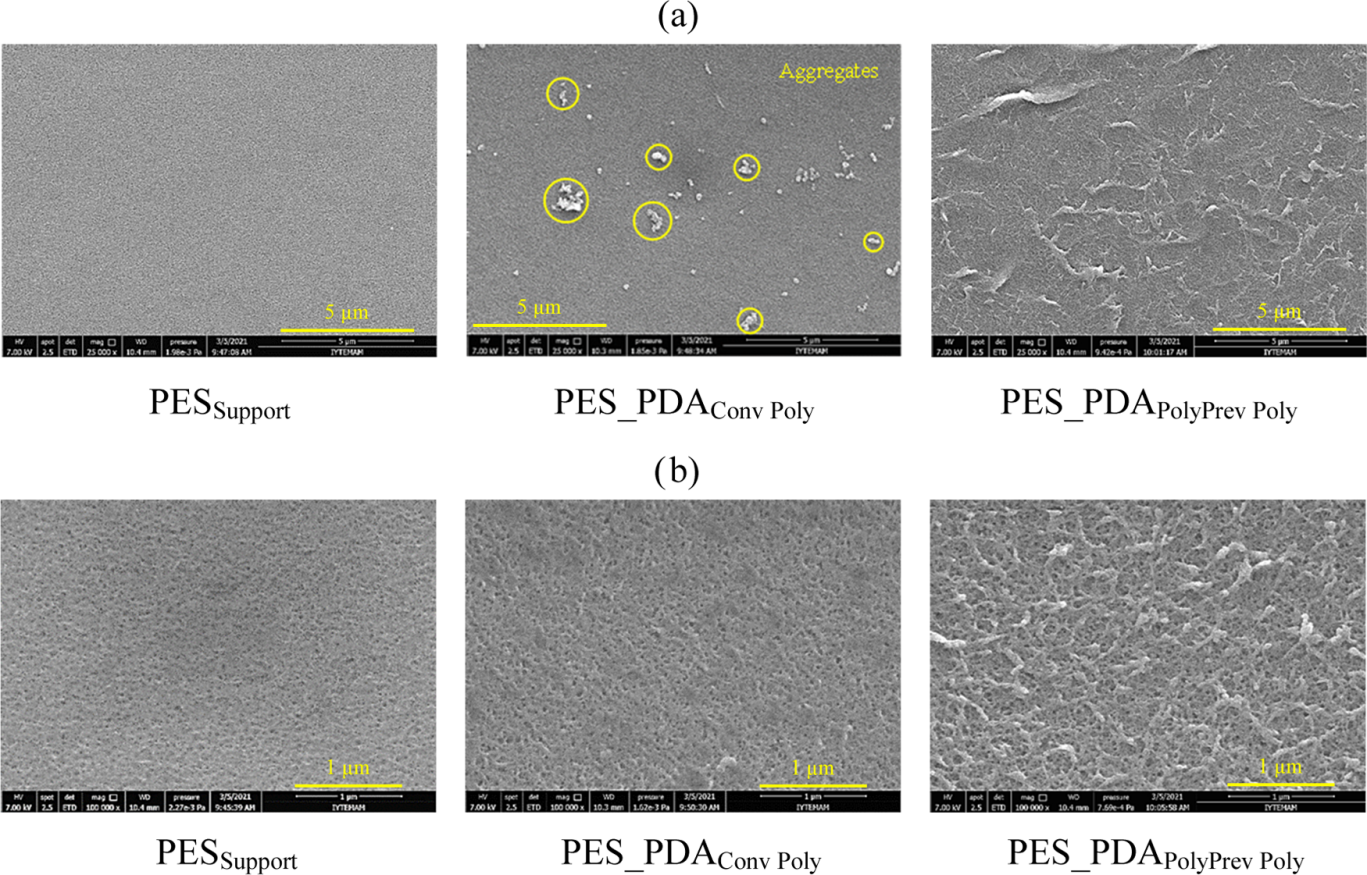
SEM micrographs of the active side of the support and PDA-coated membranes (a) 25 kX magnification and (b) 100 kX magnification.

SEM micrographs were acquired at two different magnifications (25 and 100 kX) and analyzed using *ImageJ* software to determine the membranes’ pore size (Figure 2). The PDA coating without N_2_ flow reduced the pore diameter of the PES_Support_ membrane from 27.8 ± 4.3 to 18. ± 1.8 nm. On the other hand, the average pore diameter of the PES_PDA_PolyPrev Poly_ membrane (26.4 ± 4.1 nm) was statistically the same as that of the PES_Support_ membrane (27.8 ± 4.3 nm). This result supports the hypothesis that N_2_ backflow prevents the polymerization in the pores. The measured pore size of the pristine membrane is in agreement with the value (26 nm) reported by the manufacturer.^42^ The SEM micrographs also revealed that the surface of the PES_Support_ membranes changed after being coated with PDA. Some aggregates were observed on PDA-coated membranes prepared using the conventional method (no N_2_ flow); see Figure 2. Depending on the dopamine concentration and reaction temperature, the formation of aggregates during the traditional polymerization of dopamine is inevitable.^43^ Vecchia et al. reported that dopamine begins forming oligomers at the beginning of polymerization, and the formed oligomers create seeds for growing PDA aggregates throughout the polymerization.^44^ Continuous N_2_ backflow during polymerization created a barrier between the solid–liquid interface, disrupting aggregate formation on the membrane surface. As a result, no PDA aggregates were observed on the PES_PDA_PolyPrev Poly_ membrane.

The AFM results provided in Figure S1 and Table S1 show that the PDA coating increased the surface roughness of the PES_Support_ membrane. The dopamine polymerization with the N_2_ backflow resulted in a slightly higher surface roughness than the conventional polymerization without gas flow. Continuous nitrogen feeding creates a perpendicular barrier to the membrane pores, forcing the polymer to be positioned vertically, resulting in a rougher surface.

**Figure 3:**
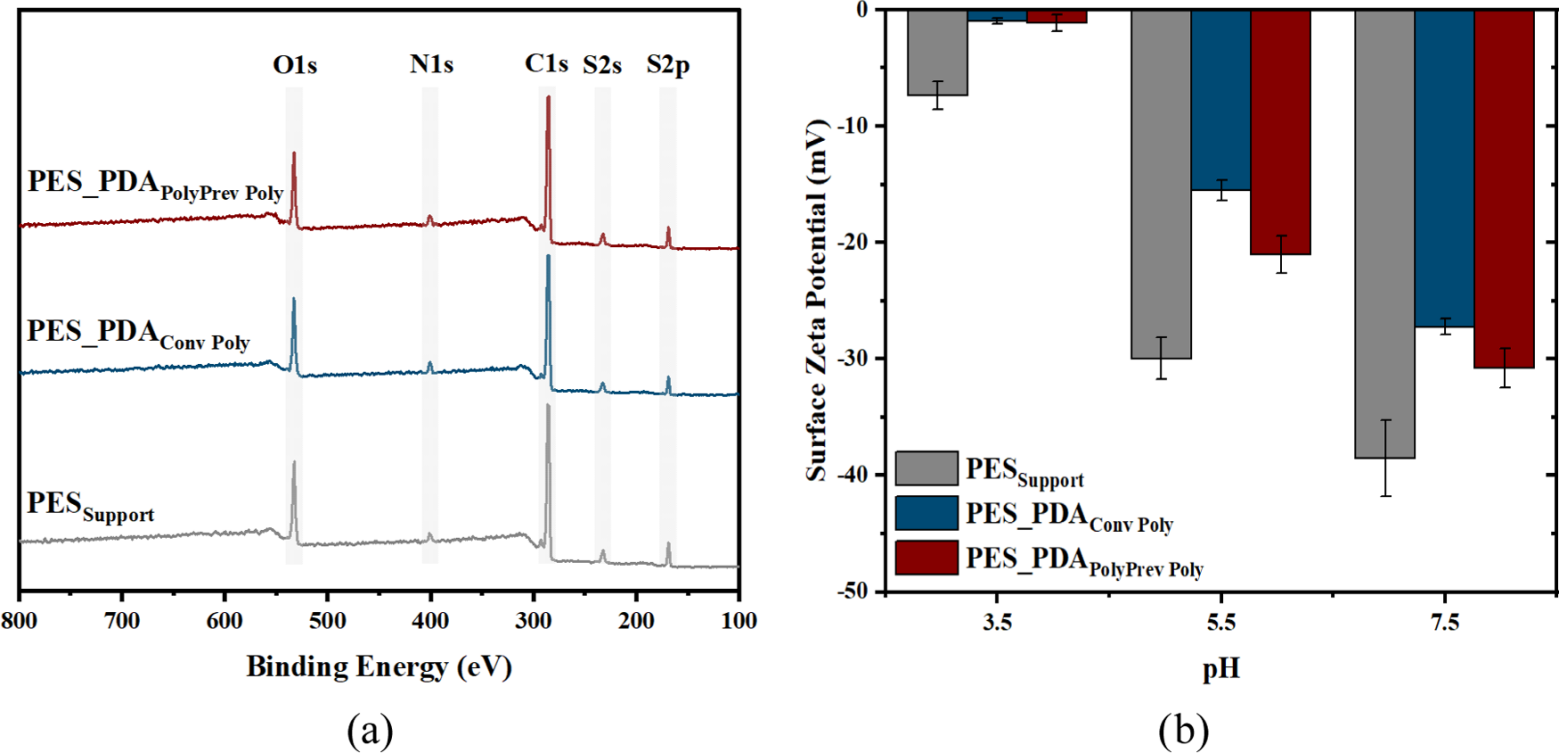
(a) XPS survey and (b) surface ζ potential of the support and PDA-coated membranes.

The surface chemistry of the membranes was analyzed by XPS, and the results are provided in Table 1 and Figure 3a. Nitrogen and sulfur signals are the characteristic indicators for the PDA and PES, respectively. The nitrogen signal in the unmodified PES_Support_ membrane comes from the pore former, polyvinyl pyrrolidone (PVP).^43^ The nitrogen atomic percentage increased for both PDA-coated membranes, while the sulfur percentage decreased, as expected. The PES_PDA_PolyPrev Poly_ membrane had a lower N/S ratio, indicating a thinner PDA layer formed on the support. This result was found in agreement with the less intense color observed in the digital images taken from the active side (topside) of this membrane (Figure 1c).

**Table 1:** Surface Elemental Composition (wt %) of the Support and PDA-Coated Membranes

membranes	C (%)	O (%)	S (%)	N (%)	N/S
PES_Support_	74.17 ± 0.82	17.31 ± 0.54	6.51 ± 0.30	2.01 ± 0.21	0.31
PES_PDA_Conv Poly_	73.73 ± 0.84	18.18 ± 0.52	4.67 ± 0.23	3.42 ± 0.41	0.73
PES_PDA_PolyPrev Poly_	74.43 ± 0.73	17.43 ± 0.61	5.39 ± 0.22	2.75 ± 0.33	0.51

The surface charge of the unmodified support and modified membranes was determined at three different pH values (Figure 3b). The unmodified PES_Support_ membrane is negatively charged due to sulfone groups in its structure.^45^ PDA coatings have primary, secondary, and tertiary amine groups.^12,46,47^ These groups are Lewis bases and owe their properties to nonbonding electron pairs. The nitrogen atoms with a lone pair of electrons in amine groups have a lower electronegativity and higher nucleophilicity than oxygen. Therefore, they act as an electron donor and tend to react with hydrogen atoms to gain a positive charge, consequently, the modified membranes carry fewer negative charges.^48,49^ This result provided additional support for the successful deposition of PDA. At pH values of 5.5 and 7.5, the PES_PDA_Conv Poly_ membrane was more protonated due to the presence of more nitrogen atoms on its surface, consistent with the XPS analysis (Table 1).

The changes in the hydrophilicity of the membranes upon PDA coating were determined using contact angle measurements (Table S2). The PES_Support_ had a water contact angle of 61.4 ± 3.7°, consistent with the literature on commercial PES UF membranes.^25,50,51^ After modification with the PDA layer, the water contact angle decreased. The PDA layer formed with conventional and PolyPrev polymerization systems improved the hydrophilicity of the support equally (PES_PDA_Conv Poly_ membrane: 44.4 ± 2.4° and the PES_PDA_PolyPrev Poly_ membrane: 46.7 ± 1.9°). An ideal surface modification technique should enhance the surface properties, such as hydrophilicity without changing the permeance and selectivity of the membrane. In this respect, the results suggest that this protocol that featured dopamine polymerization with N_2_ backflow is promising to use for our next set of experiments, which aim to codeposit dopamine with an antibacterial agent.

### 3.2 Characterization and Performance of the CTAB-Functionalized Membranes Prepared Using the PolyPrev System

PDA coatings do not show sufficient antibacterial activity against Gram-positive and Gram-negative bacteria.^15−17^ Codeposition of an antibacterial agent with dopamine is an effective strategy for making the surface antibacterial. The main challenge is to achieve a thin antibacterial coating layer to minimize the mass transfer resistance. We overcame this challenge by choosing a low-molecular-weight active agent, CTAB. The codeposition of CTAB with dopamine was investigated on quartz slides^52^ but has never been utilized to improve the anti-biofouling properties of membranes.

**Figure 4:**
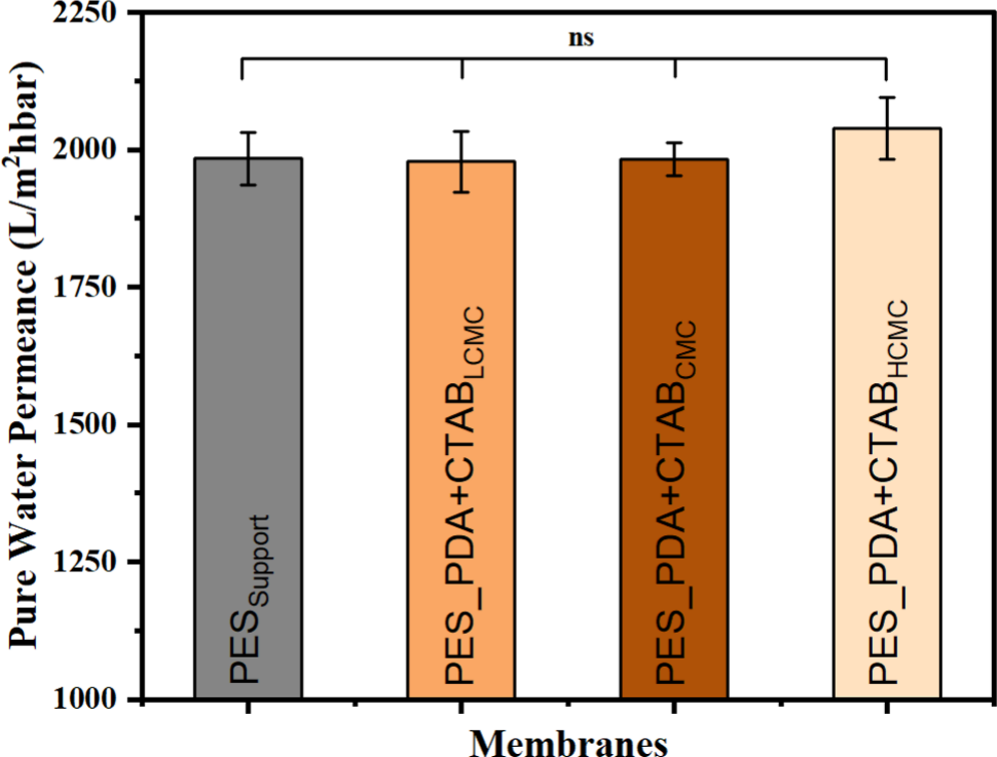
Pure water permeance of the support and codeposited membranes as a function of CTAB concentration. ns represents no statistical difference (*p* > 0.05) in pure water permeance of all of the membranes.

The suitability of using a concentration of CTAB below, at, and above the CMC for codeposition with dopamine was first evaluated via the PWP measurements. As shown in Figure 4, the modified membranes exhibited statistically equivalent PWP to the PES_Support_ membrane. The results suggest that N_2_ backflow mitigated the solution intrusion phenomenon, and the presence of CTAB did not block the pores.

Table S3 compares the performance of CTAB/dopamine codeposition with different modification methods. Prior studies have two significant disadvantages: (1) the long modification times needed to change the surface characteristics and (2) significant flux reduction upon modification. Compared to the surface modification techniques reported in Table S3, our method offers a greener membrane fabrication process due to fewer reaction steps, avoiding extra solvent use, and eliminating the need for a UV source or vacuum filtration for modification. Additionally, because we were able to demonstrate a maintained flux after our modification protocol, we again offer a greener separation process with lower energy required to achieve the equivalent filtration. In contrast to our single-step protocol, Weinman et al. coated the same commercial membrane used in the current study with a zwitterionic polymer, poly(2-((2-hydroxy-3-(methacryloyloxy) propyl) dimethyl ammonio) acetate) (poly(CBOH)) using a multistep procedure.^51^ First, the PES membrane was treated using a photoinitiator for 4 h before being exposed to UV light for 4.5 h to graft CBOH. The poly(CBOH) coating reduced the bacterial deposition by order of magnitude versus the unmodified membrane; however, it caused a decrease in the average water permeance of the membrane from 915 to 770 L/m^2^hbar. Zhang et al. grafted a zwitterion polyampholyte hydrogel onto a 134 kDa in-house fabricated PES membrane^53^ using a multistep process. The water permeance of the pristine PES membrane was 133 ± 4 L/m^2^hbar, and it decreased to 93 ± 6 L/m^2^hbar and 70 ± 5 L/m^2^hbar after the zwitterionic polyampholyte hydrogel grafting and loading with the GO nanosheets, respectively. In the work by Xueli et al., the PSF UF membranes were modified by UV-grafting for only 30 min, but their flux decreased by 36%.^54^

**Figure 5:**
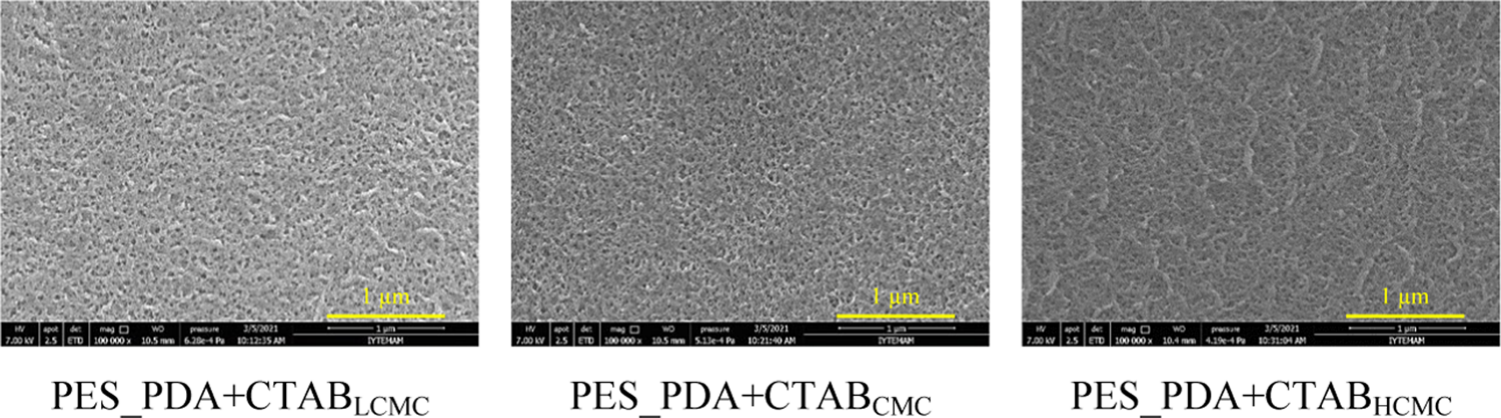
SEM micrographs of the active surface side of the codeposited membranes (100 kX magnification).

**Figure 6:**
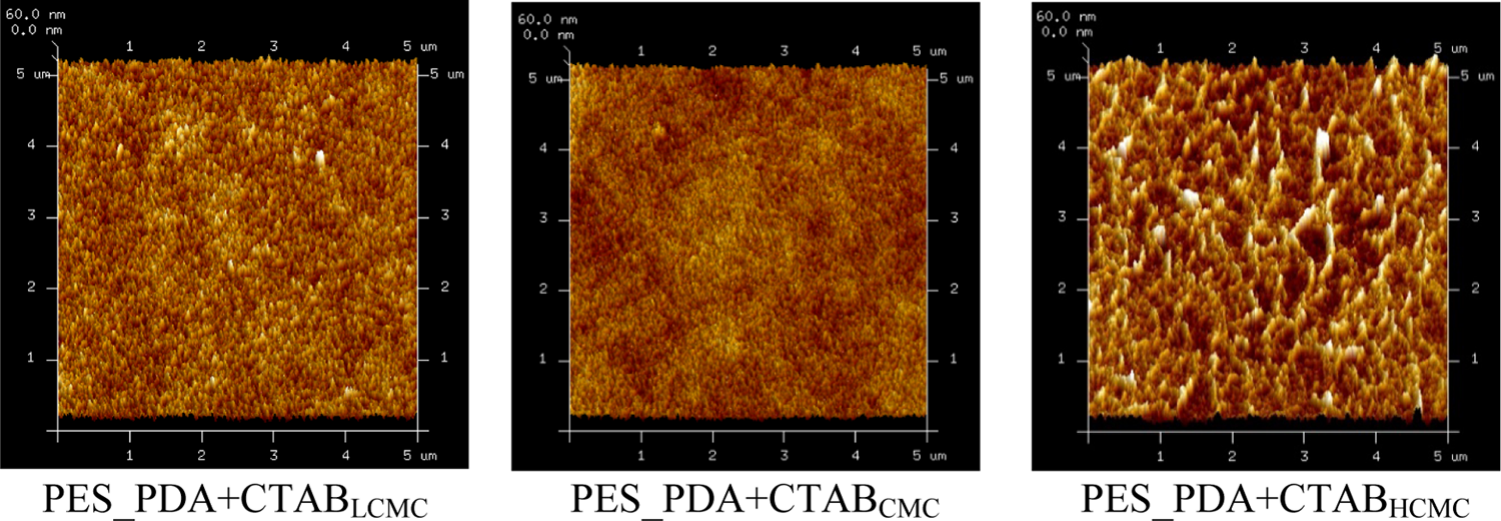
AFM images of the codeposited membranes.

**Scheme 2:**
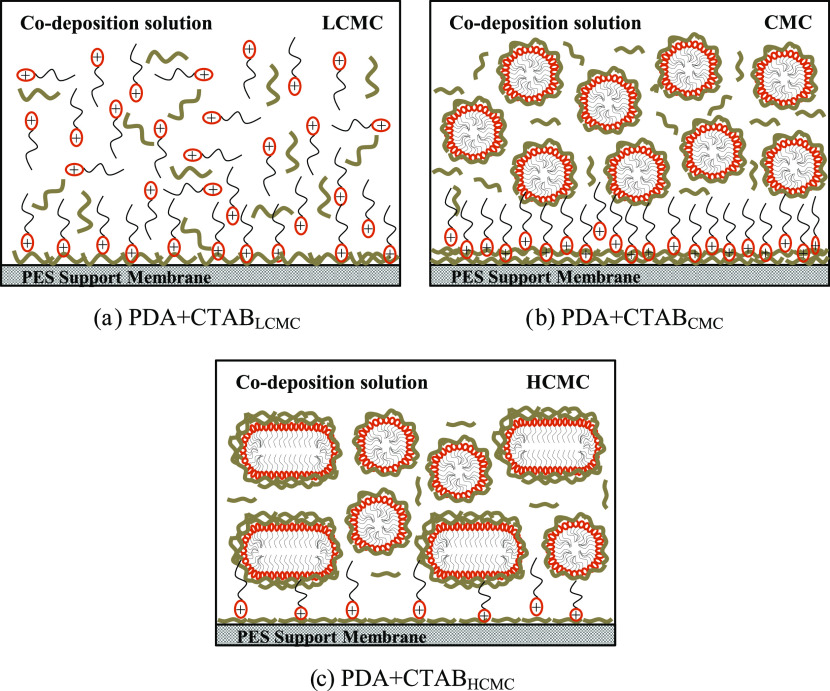
Effect of CTAB Concentration on the Morphology of the Codeposition Coating in the (a) PDA + CTAB_LCMC_, (b) PDA + CTAB_CMC_, and (c) PDA + CTAB_HCMC_ solutions

SEM micrographs were used to determine the average pore diameters of our codeposited membranes (PES_PDA + CTAB_LCMC_ (28.1 ± 3.8 nm), PES_PDA + CTAB_CMC_ (28.4 ± 4.7 nm), PES_PDA + CTAB_HCMC_ (27.9 ± 3.5 nm)), which were statistically equivalent to that of the support membrane (PES_Support_ (27.8 ± 4.3 nm)) (Figure 5). AFM images and surface roughness of the codeposited membranes are shown in Figure 6 and Table 2. Previous studies reported that the surface morphology and roughness of coated membranes are affected by the concentration of codeposited molecules, polymerization time, pH value, and temperature.^15,17,55−60^ Similarly, we found that the surface roughness of membranes changed in response to the concentration of CTAB used. The PDA-only coating increased the roughness of the pristine membrane because PDA deposition is known to form aggregates due to noncovalent bonding between PDA molecules, such as π–π stacking and hydrogen bonds. On the other hand, CTAB concentrations that are below and at the CMC suppress PDA from forming large aggregates by interrupting those hydrogen bonds and π–π stacking interactions. Hence, the roughness of the PDA–CTAB layer is reduced (Table S1). A similar result was observed by other groups where the codeposition of PDA with polyethyleneimine (PEI) and the polyethylenimine-quaternized derivative lowered the roughness of PDA coatings.^15,17,59−61^ Above the CMC, the shape of CTAB molecules changed from spherical to large-sized cylindrical micelles^62^ (Scheme 2), resulting in the formation of rough surfaces. The surface roughness of the PES_PDA + CTAB_CMC_ membrane was statistically equivalent to the roughness of the PES support because the highest loading of CTAB occurred at the CMC where the interactions between the PDA and CTAB were the strongest.

**Table 2:** Surface Properties of the Codeposited Membranes

membranes	*R*_a_ (nm)	*R*_q_ (nm)	pore diameter (nm)
PES_PDA + CTAB_LCMC_	5.52 ± 0.01	6.96 ± 0.02	28.12 ± 3.81
PES_PDA + CTAB_CMC_	4.74 ± 0.38	6.01 ± 0.47	28.43 ± 4.74
PES_PDA + CTAB_HCMC_	6.32 ± 0.64	7.94 ± 0.75	27.91 ± 3.51

**Figure 7:**
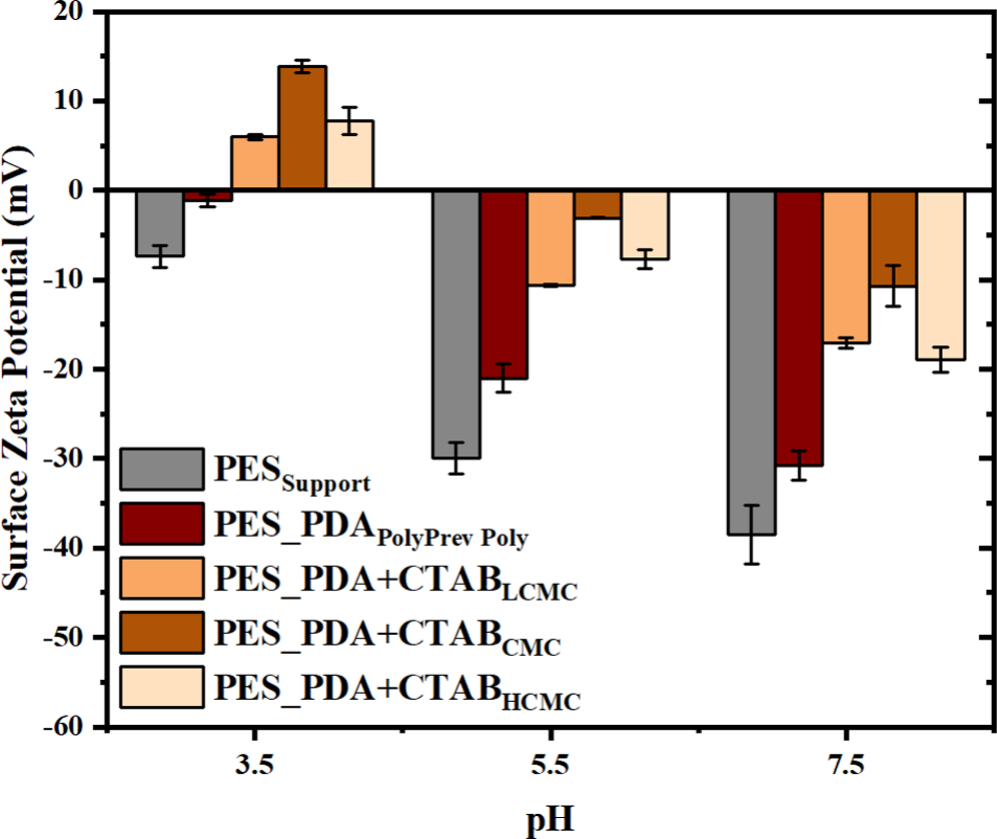
Surface ζ potential of the codeposited membranes.

The surface hydrophilicities of the PDA-coated membranes decreased after codeposition with CTAB and were 65.3 ± 1.1, 67.7 ± 1.4, and 63.9 ± 1.8° for the PES_PDA + CTAB_LCMC_, PES_PDA + CTAB_CMC_, and PES_PDA + CTAB_HCMC_ membranes, respectively, consistent with the literature.^40^ This is an expected result because the hydrophilic quaternary ammonium head group in the structure of CTAB interacts with the catechol group in the PDA, and the hydrophobic tail in the CTAB structure becomes free. This free tail makes the surface of the codeposited membranes more hydrophobic than those of pure PDA-coated membranes. Figure 7 shows that all of the codeposited membranes were more protonated than the solely PDA-functionalized membrane (PES_PDA_PolyPrev Poly_) due to the positively charged quaternary ammonium head group on the surface. The surface charge gave indirect information about the loading of CTAB on the surface. The PES_PDA + CTAB_CMC_ membrane had the lowest negative charge at pH values 5.5 and 7.5 and the highest positive charge at a pH of 3.5.

XPS was used to determine the elemental compositions of the membranes, and the results are provided in Table 3. Nitrogen is a characteristic signal for both CTAB and dopamine, whereas the elements, C and O were also detected. The N content of the support increased after the introduction of dopamine and the dopamine/CTAB layer on the surface. The N 1s spectra were analyzed in detail to prove the presence of CTAB in the dopamine layer (Figure S3). The characteristic peaks at ∼399.6 and ∼400.2 eV binding energies were attributed to the C–N group and the aromatic C–N and C=N groups. These two groups are in the structure of dopamine and the pore former in the support membrane. On the other hand, the peak at ∼402.5 eV belongs to the quaternary ammonium group;^17,55,56^ thus, it was observed only in the dopamine layer codeposited with CTAB. The peak areas under the deconvoluted curves were calculated and used to quantify the groups in the structure. As shown in Table 4, the highest quaternary ammonium amount was found in the PES_PDA + CTAB_CMC_ membrane, which explained why the highest positive charge was observed for this membrane (Figure 7).

**Table 3:** Surface Elemental Compositions (wt %) of the Support and Codeposited Membranes

membranes	C (%)	O (%)	S (%)	N (%)	N/S
PES_Support_	74.17 ± 0.81	17.31 ± 0.51	6.51 ± 0.31	2.01 ± 0.20	0.31
PES_PDA_PolyPrev Poly_	74.43 ± 0.73	17.43 ± 0.62	5.39 ± 0.21	2.75 ± 0.32	0.51
PES_PDA + CTAB_LCMC_	75.57 ± 0.70	16.55 ± 0.60	4.81 ± 0.20	3.07 ± 0.21	0.64
PES_PDA + CTAB_CMC_	75.69 ± 0.82	16.11 ± 0.41	4.58 ± 0.24	3.62 ± 0.34	0.79
PES_PDA + CTAB_HCMC_	76.16 ± 0.64	15.72 ± 0.53	5.48 ± 0.23	2.64 ± 0.32	0.48

**Table 4:** Peak Areas under the Deconvoluted Curves

membranes	C–N group at ∼399.6 eV	aromatic C–N and C=N groups at ∼400.2 eV	quaternary ammonium group at ∼402.5 eV
PES_Support_	785	2412	
PES_PDA_PolyPrev Poly_	1364	1485	
PES_PDA + CTAB_LCMC_	1133	1150	400
PES_PDA + CTAB_CMC_	3852	726	519
PES_PDA + CTAB_HCMC_	85	1625	207

**Figure 8:**
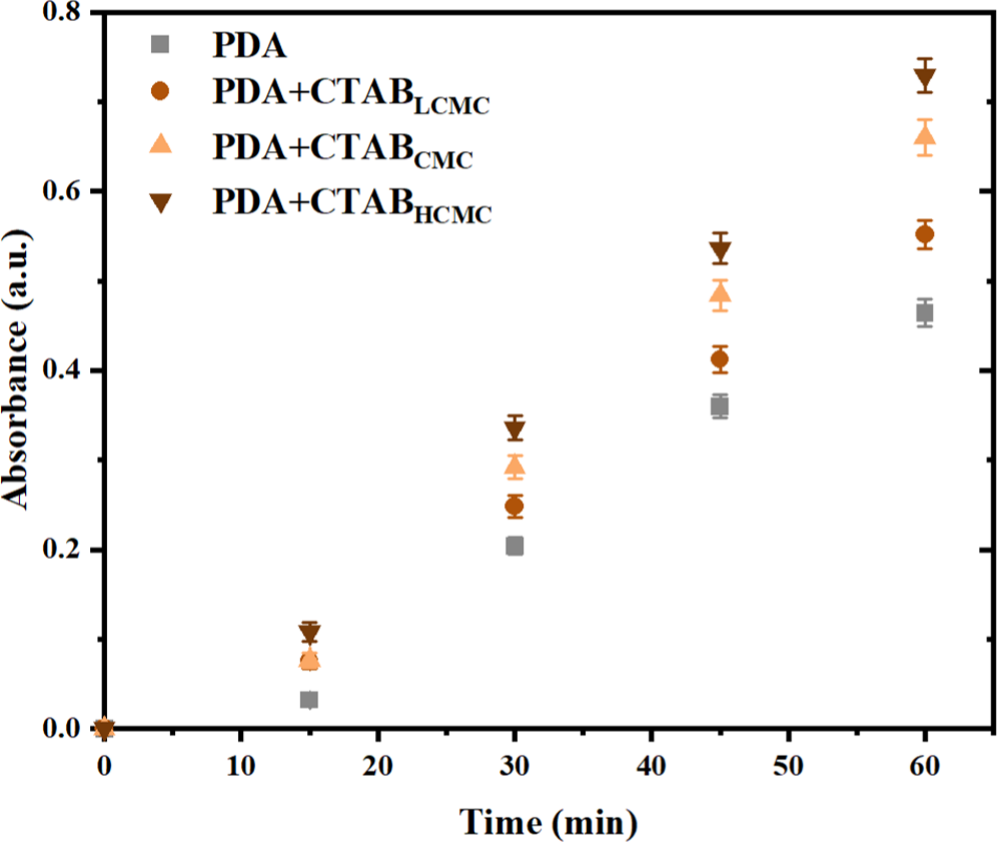
Absorbance of PDA as a function of CTAB concentration in the liquid phase.

To understand why the lowest CTAB loading was observed above the CMC, we evaluated the dopamine polymerization in the liquid phase by measuring the absorbance (at 420 nm) of the dopamine solution taken from above the membrane surface.^63^ As shown in Figure 8, in the presence of CTAB, the extent of liquid-phase polymerization increased according to the increased absorbance values, which demonstrated that CTAB acts as a template for polymerization.^57^ We also quantified the inhibition effect of CTAB on the PDA deposition rates by measuring the thickness of deposited layers on silicon wafers. After 72 h of coating time, the thickness of the PDA layer decreased by half at the CMC of the CTAB, and we could not detect a deposited layer when the CTAB concentration was above its CMC (Table 5). The highest liquid-phase polymerization and the inhibition of the deposition rates were observed at a CTAB concentration above its CMC. The templating effect of CTAB for initiating polymerization in the liquid phase is proportional to its surface area. As shown in Scheme 2, the CTAB molecules have the highest surface area above the CMC. Additionally, an increased positive charge density with the CTAB concentration enhanced the interaction between dopamine and CTAB, hence, the polymerization in the liquid phase. Furthermore, excess CTAB molecules above its CMC make the PDA aggregates stable in the deposition solution leading to a low deposition rate. Previous studies have also reported the inhibition of PDA deposition rates in the presence of ionic surfactants^52^ and polymers.^64^ Nonionic surfactants and polymers did not affect liquid-phase polymerization and deposition rates. Our results suggest that the CTAB concentration in the codeposition solution should not exceed its CMC to minimize the initiation of polymerization in the liquid phase and prevent the stability of formed PDA aggregates in the solution. Based on the results, it can be concluded that the lowest positive charge at neutral pH (Figure 7) and the lowest quaternary ammonium group (Table 4) observed for the PES_PDA + CTAB_HCMC_ membrane are due to the dominance of liquid-phase polymerization over interfacial polymerization of dopamine when the CTAB in the dopamine solution was above its CMC.

**Table 5:** Thicknesses of Dry Coatings on Crystalline Silicon Wafers

	coating thickness (nm)	
membranes	24 h	72 h
PDA	24.24 ± 1.91	44.03 ± 1.11
PDA + CTAB_LCMC_	21.21 ± 0.94	31.51 ± 0.10
PDA + CTAB_CMC_	18.53 ± 0.54	21.34 ± 1.52
PDA + CTAB_HCMC_	a	a

a The thickness could not be measured.

### 3.3 Anti-Biofouling Assessment of Membranes

**Figure 9:**
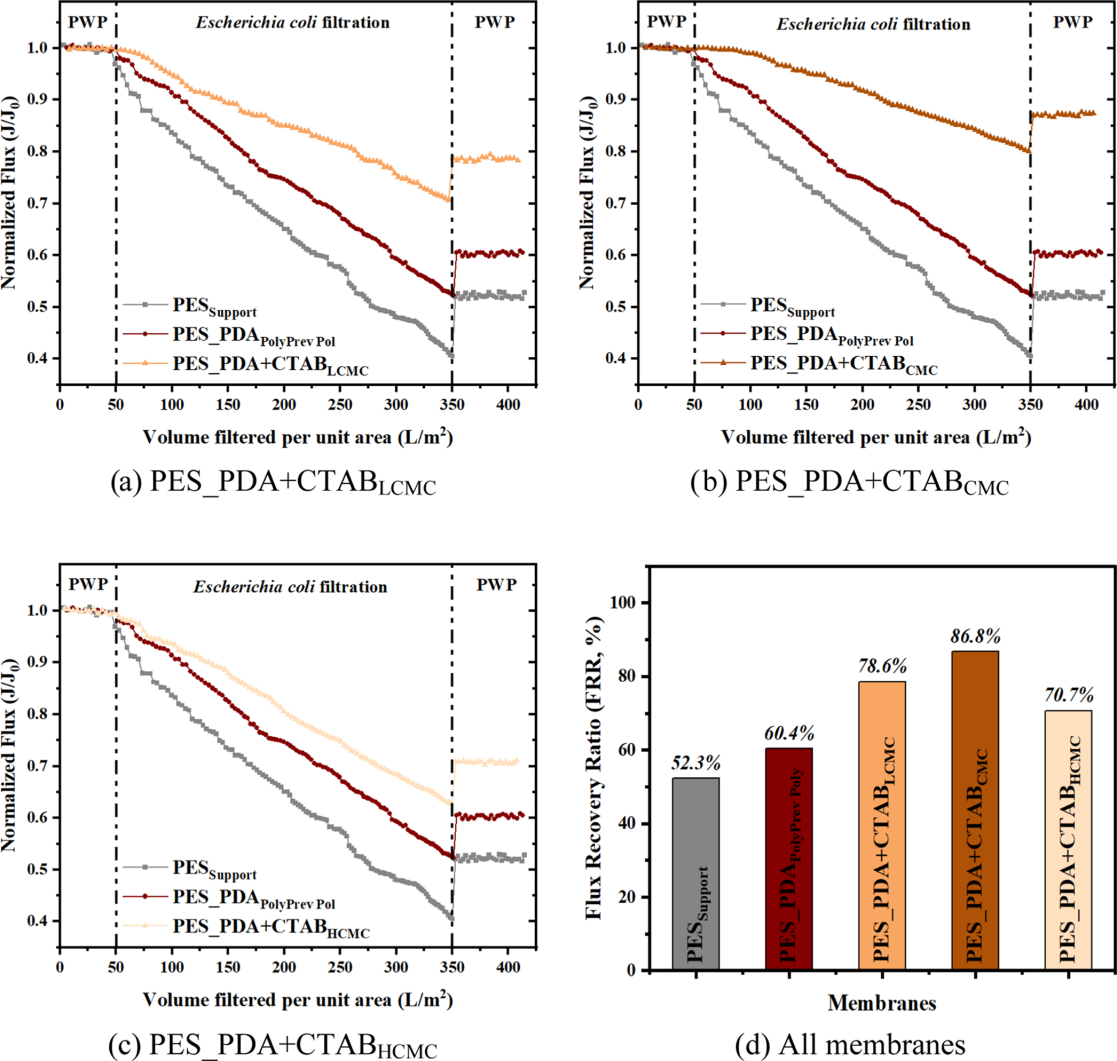
Normalized flux of the support, PDA-coated, and (a) PES_PDA + CTAB_LCMC_, (b) PES_PDA+CTAB_CMC_, and (c) PES_PDA + CTAB_HCMC_ codeposited membranes as a function of the volume filtered per unit area during *E. coli* filtration and (d) FRR of all membranes after *E. coli* filtration. Transmembrane pressure (TMP) applied to the membranes for the bacterial filtration was 0.3 bar.

**Figure 10:**
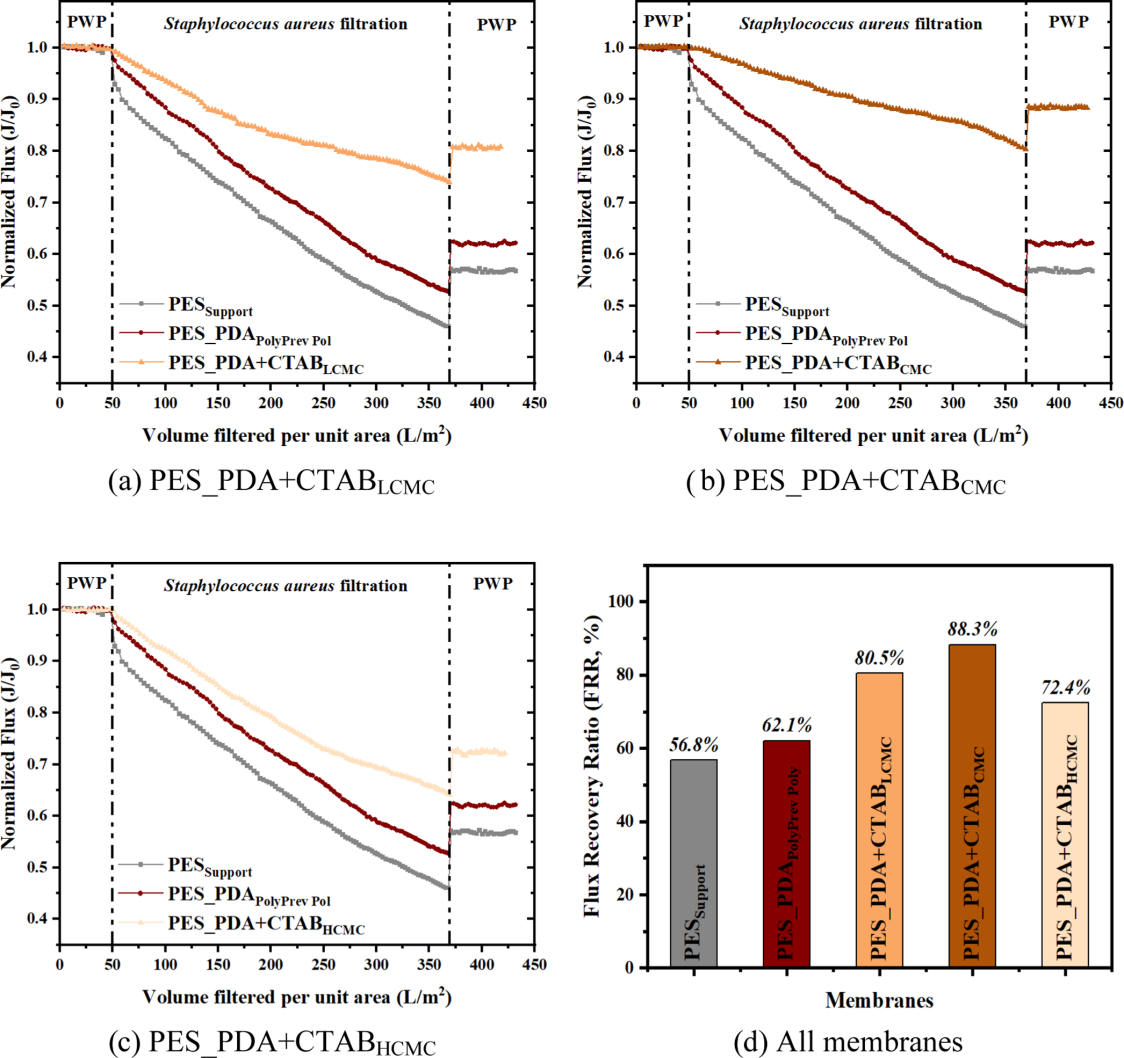
Normalized flux of the support, PDA-coated, and (a) PES_PDA + CTAB_LCMC_, (b) PES_PDA + CTAB_CMC_, and (c) PES_PDA + CTAB_HCMC_ codeposited membranes as a function of volume filtered per unit area during *S. aureus* filtration and (d) FRR of all membranes after *S. aureus* filtration. Transmembrane pressure (TMP) applied to the membranes for the bacterial filtration was 0.3 bar.

The anti-biofouling behavior of the support (PES_Support_), PDA-coated (PES_PDA_PolyPrev Poly_), and codeposited (PES_PDA + CTAB_LCMC_, PES_PDA + CTAB_CMC_, PES_PDA + CTAB_HCMC_) membranes were evaluated by conducting dynamic filtration experiments using Gram-positive (*S. aureus*) and Gram-negative (*E. coli*) bacterial suspensions using the dead-end filtration unit. As shown in Figures 9 and 10, the PES_Support_ membrane exhibited the greatest flux decline and the lowest flux recovery ratio (FRR), which are measures of the biofouling resistance of a membrane. After coating with a PDA layer, the biofouling resistance of the PES_PDA_PolyPrev Poly_ increased. The improvement can be attributed to the anti-adhesion property that resulted from enhanced hydrophilicity and the reduced ζ potential of the coated PDA layer.

The codeposition of dopamine with all CTAB concentrations caused a lower flux decline than the support and PDA-coated membranes during bacterial filtration. The higher biofouling resistance of the codeposited membranes resulted from the strong antibacterial activity of CTAB.^31^ The antiadhesive property of a surface can only reduce the initial bacterial adsorption. On the other hand, the antibacterial surface attacks, disperses, or suppresses the activity of attached organisms. Additionally, the lower electrical charge^4^ of the CTAB-containing PDA membranes contributed to its lower biofouling propensity. The roughness of the unmodified and all modified membranes was small enough to prevent penetration of the bacteria into the valleys (Tables S1 and S2) and thus, it did not affect the biofouling tendency of the membranes during our experiments.

Among the codeposited membranes, the PES_PDA + CTAB_CMC_ membranes showed the lowest flux decline and the highest FRR; thus, the best anti-biofouling performance against both microbes. This observation was directly related to this membrane’s highest loading of CTAB (Table 4). The PDA coating increased the FRR of the PES support from 52.3 to 60.4% following backwashing after the *E. coli* filtration. On the other hand, codeposition of dopamine with CTAB at its CMC increased the FRR to 86.8%.

### 3.4 Antibacterial Assessment of Membranes

**Figure 11:**
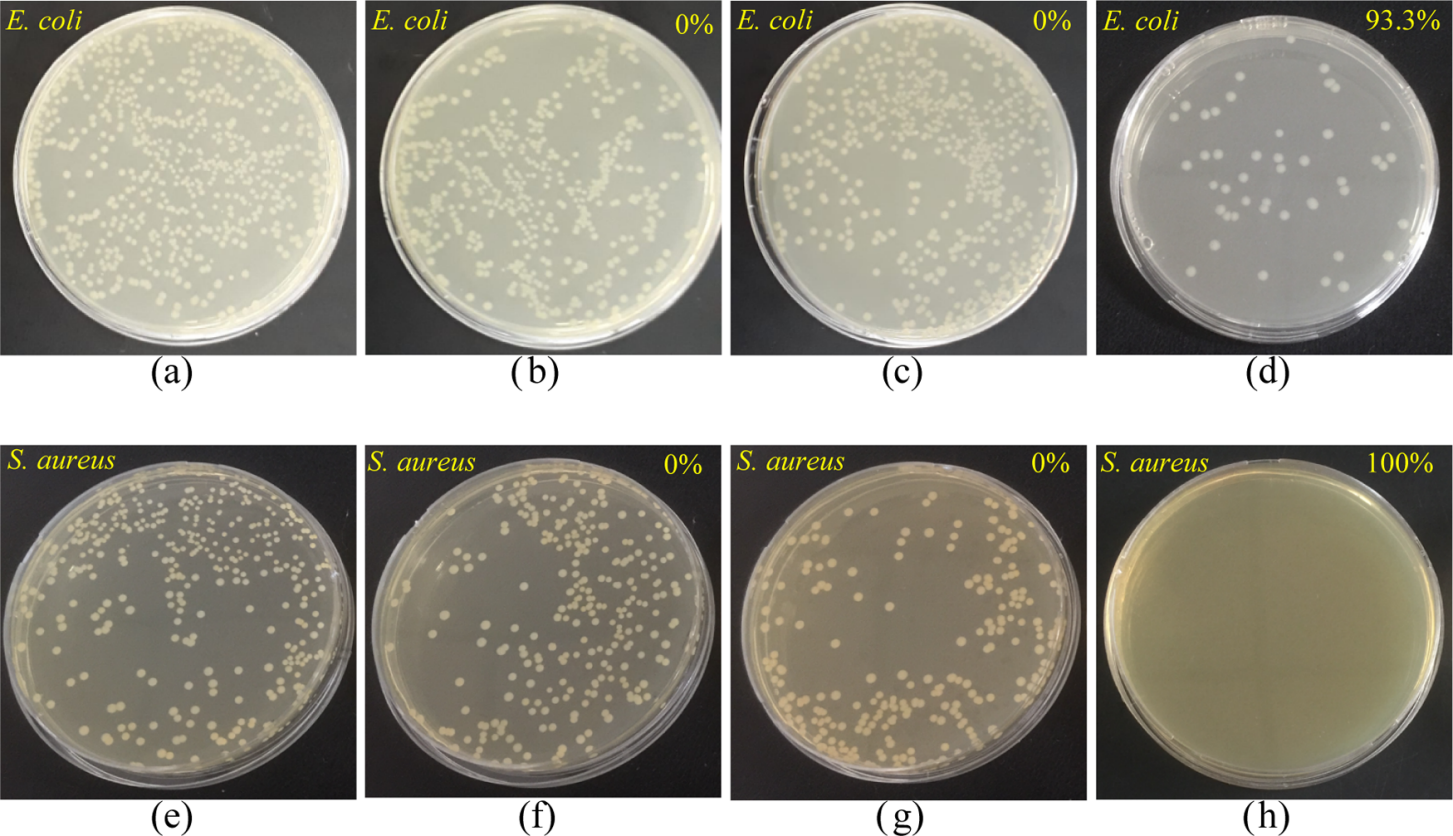
Antibacterial activity of the (a, e) control (incubated medium without membranes), (b, f) PES_Support_, (c, g) PES_PDA_PolyPrev Poly_, and (d, h) PES_PDA + CTAB_CMC_ membranes (*E. coli* and *S. aureus* suspensions were diluted 10^2^-fold before spreading on agar plates).

We also determined the antibacterial activity of the membranes against Gram-positive (*S. aureus*) and Gram-negative (*E. coli*) bacteria using the ASTM-E2180 colony-counting method. As expected, the PES_Support_ and the PES_PDA_PolyPrev Poly_ membranes did not exhibit any antibacterial activity against either bacteria due to the absence of any active chemical moieties (Figure 11). On the other hand, once CTAB was present on the membranes, it imparted excellent antibacterial activity. CTAB’s antibacterial activity comes from the disruption of the bacteria’s negatively charged cell wall with its positively charged head groups leading to leakage of substances in the cell.^65^Table 6 lists the antibacterial activity of membranes modified with different bactericidal agents. Since the membrane area was not reported in most studies, a direct comparison of antibacterial activities achieved at the end of a 24 h incubation period was not possible. However, a few remarks can still be noted from the table. For example, Wang et al. reported 100 and 99.93% *E. coli* inactivation rates by exposing their membrane to 10 times lower bacterial concentrations ((1.5 × 10^4^ CFU/cm^2^)^66^ and (2.4 × 10^3^ CFU/cm^2^)^67^) compared to our concentration (11.7 × 10^4^ CFU/cm^2^). In our recent studies, we reported excellent antibacterial activities for polysulfone–sulfonated polyethersulfone (PSF–SPES)^40^ and citric acid-doped polyaniline^68^-based UF membranes. Interestingly, when used at the CMC, CTAB in PSF–SPES and dopamine layers resulted in similar antibacterial activities even though these membranes were prepared using different protocols.

**Table 6:** Antibacterial Activity of PES UF Membranes from the Literature^a^

				volume		concentration (CFU/mL)		antibacterial rate (%)		
membranes	modification method	contact time	contact area (cm^2^)	*E. coli*	*S. aureus*	*E. coli*	*S. aureus*	*E. coli*	*S. aureus*	refs
GO-p-PES	UV-graft	3 h	1.54	100 μL		10^5^		80.0		(53)
HNTs-CS@Ag/PES	mixed matrix	24 h		5 mL	5 mL	10^6^	10^6^	94.0	92.6	(69)
HPEI-GO/PES	mixed matrix	24 h		5 mL		10^6^		74.9		(70)
PSF/PES-AM-VT 1.0	mixed matrix	24 h	3					92.3		(71)
PES/SPSF/GO	mixed matrix	18 h		45 mL				90.0		(72)
rGO-ZnO/PES	mixed matrix	3 h	1.13	100 μL	100 μL	10^6^	10^6^	95.0	<10	(73)
ZGO-NH/PES	mixed matrix	6 h	6	10 mL	10 mL	10^6^	10^6^	81.1	85.7	(74)
PES/TPQP-Cl	mixed matrix	12 h	4	10 mL				65.0		(75)
PES_PDA + CTAB_CMC_	codeposition	24 h	9	300 μL	300 μL	3.5 × 10^6^	4.2 × 10^6^	93.3	100	this work

a GO: Graphene oxide, PES: polyethersulfone, HNTs-CS@Ag: halloysite nanotube–chitosan–Ag nanoparticles, HPEI-GO: hyperbranched poly(ethyleneimine-graphene) oxide, PSF: polysulfone, AM: capsaicin-mimic *N*-(5-methyl acrylamide-2,3,4 hydroxy benzyl) acrylamide, VT: vinyl triethylene (b-methoxy ethoxy) silane, SPSF: sulfonated polysulfone, rGO: reduced graphene oxide, ZnO: zinc oxide, ZGO-NH: zeolitic imidazole framework-8 decorated with graphene oxide functionalized with amino groups, TPQP-Cl: (4,6-trimethoxyphenyl) polysulfone–methylene quaternary phosphonium chloride, and CTAB_CMC_: cetyltrimethylammonium bromide at the critical micelle concentration.

### 3.5 Long-Term Chemical Stability of the CTAB in the Membrane

The excellent anti-biofouling properties of the CTAB-containing membranes resulted from the strong antibacterial activity provided by CTAB. Thus, it is important that the CTAB-rich coating is stable over a long period of time. CTAB is ionically bound to PDA through electrostatic interactions between positively charged quaternary ammonium (NR_4_^+^) and the negatively charged catechol groups. The harshest environment for an ionic bond is high salt concentration since the salt ions weaken the electrostatic interaction by increasing the distance between the charged groups. Based on this fact, we used a very high salt concentration (1 M NaCl) to test the strength of the ionic bond between CTAB and PDA. Our experiment focused on the membrane that displayed the strongest antibacterial and anti-biofouling performances (PES_PDA + CTAB_CMC_). The membrane was stored in 1 M NaCl (pH = 6.8) solution for 3 months at room temperature (25 °C). An accurate method for determining the leached CTAB in water could be measuring the N element amount with total organic carbon (TOC) analysis. However, this measurement can be misleading since the N element also comes from the water-soluble PVP used as a pore former in the support. As an alternative, ζ potential measurements were used because they are a good indicator of the stability of the CTAB since the presence of CTAB significantly alters the charge of the support and the membrane functionalized with PDA alone (Table 7). The results showed that after 3 months of storage in 1 M NaCl solution, the PES_PDA + CTAB_CMC_ membrane had ζ potential values equivalent to its fresh counterparts proving strong electrostatic interactions between CTAB and PDA. Our results suggest that CTAB codeposited with dopamine enhanced the biofouling resistance of the commercial PES_Support_ and remained stable in the deposited layer. While further experiments that confirm the biological activity of the membranes after long-term storage would be interesting, they are beyond the scope of this study.

**Table 7:** Surface ζ Potential Measurements of the Fresh PES_Support_, PES_PDA_PolyPrev Poly_, and PES_PDA + CTAB_CMC_ Membranes and the ζ Potential of the PES_PDA + CTAB_CMC_ after 3 Months of Exposure to 1 M NaCl Solution

	surface ζ potential (mV)		
membranes	pH 3.5	pH 5.5	pH 7.5
PES_Support_	–7.38 ± 1.21	–29.94 ± 1.80	–38.54 ± 3.29
PES_PDA_PolyPrev Poly_	–1.13 ± 0.73	–21.03 ± 1.60	–30.78 ± 1.67
PES_PDA + CTAB_CMC_	13.84 ± 0.71	–3.07 ± 0.10	–10.72 ± 2.27
PES_PDA + CTAB_CMC_ exposed to 1 M NaCl for 3 months	13.82 ± 1.22	–3.66 ± 0.57	± 10.42 ± 3.17

Previous studies reported that the PDA layer has strong stability in acidic, neutral, and weak alkaline environments but can quickly be destroyed by a strongly alkaline solution (pH > 12).^76^Figures S4,S5 show the UV absorbance values of PDA released from the coated surface and digital pictures of the membranes after immersing them in 0.1 M NaOH solution (pH = 13). The presence of CTAB reduced the absorbance of the PDA in the eluent by half. Improved stability of the codeposited layer relies on the strong electrostatic interaction between CTAB and PDA. In the literature, the codeposition of dopamine with appropriate organic^17,77−80^ or inorganic molecules^81,82^ has been thoroughly demonstrated as an approach to increase the PDA layer’s stability in strongly alkaline solutions.

## 4 Conclusions

We have demonstrated a facile surface modification that enhances the biofouling resistance of UF membranes. The approach is based on the codeposition of dopamine with a low molecular weight, strong antibacterial surfactant, CTAB, under N_2_ backflow. The PDA layer alone improved the biofouling resistance of the PES_Support_ through enhanced hydrophilicity. Importantly, the presence of CTAB imparted strong anti-biofouling properties against both Gram-negative and Gram-positive microorganisms. The concentration of CTAB in the dopamine solution significantly influenced the deposition rate and the biofouling propensity of the membranes. Among three CTAB concentrations (< CMC, = CMC, CMC), the lowest flux decline and the highest FRR were observed when the PDA was functionalized with CTAB at the CMC. Above the CMC of CTAB, the liquid-phase polymerization became dominant over interfacial polymerization.

Our results demonstrate that the codeposition protocol proposed in this study could be used to develop biofouling-resistant UF membranes without compromising the pore size and the water flux of the support. The commercial PES UF support membrane chosen in this study is commonly used for MBR applications. In submerged MBR applications, the best performance is achieved when the antibacterial agent in the membrane only kills bacteria on the membrane surface, and the release of antimicrobial agents into the reactor is prevented. Thus, we suggest that the membranes functionalized with CTAB at the CMC concentration hold great potential in submerged MBRs because CTAB provides strong antibacterial activity via direct contact with bacteria.

## Supporting Information Available

Polymerization methods; AFM, SEM, and XPS analysis; absorbance value of released PDA; digital images of membranes after stability; surface properties and contact angle measurements; and membrane characteristics and performance (PDF)
