# Supplementary material for: Biofouling-Resistant Ultrafiltration Membranes via Codeposition of Dopamine and Cetyltrimethylammonium Bromide with Retained Size Selectivity and Water Flux
Source: ACS Appl Mater Interfaces. 2022 Aug 10;14(33):38116–31. doi: 10.1021/acsami.2c05844 (PMC9412966; doi:10.1021/acsami.2c05844)
Supplement: Supplementary file 1 — am2c05844_si_001.pdf [file am2c05844_si_001.pdf]

# Supporting Information

# Biofouling-Resistant Ultrafiltration Membranes via Codeposition of Dopamine and Cetyltrimethylammonium Bromide with Retained Size Selectivity and Water Flux

Aydın Cihanoğlu[1,2], Jessica D. Schiffman[2]*, Sacide Alsoy Altinkaya[1]*

[1] *İzmir Institute of Technology, Faculty of Engineering, Department of Chemical Engineering, 35430 Urla-İzmir, Turkey*

[2] *Department of Chemical Engineering, University of Massachusetts Amherst, Amherst, Massachusetts 01003-9303, United States*

***Corresponding authors (E-mail: sacidealsoy@iyte.edu.tr; schiffman@ecs.umass.edu)***

## Table of Contents

Scheme S1. Polymerization methods used in the study. S2

Figure S1. AFM images of the support and PDA coated membranes. S3

Figure S2. SEM images of active surface side of the co-deposited membranes. S3

Figure S3. High-resolution XPS spectra of the N1s region for the membranes. S4

Figure S4. The absorbance of PDA released from the membrane surface after 6 and 24 h. S5

Figure S5. Digital images of the top active side of the membranes after alkaline stability. S5

Table S1. Surface properties of the support and PDA coated membranes. S6

Table S2. Contact angle measurements of the support, PDA coated and co-deposited membranes. S6

Table S3. Membrane characteristics and performance after various surface modifications, as reported in the literature S7

References S8

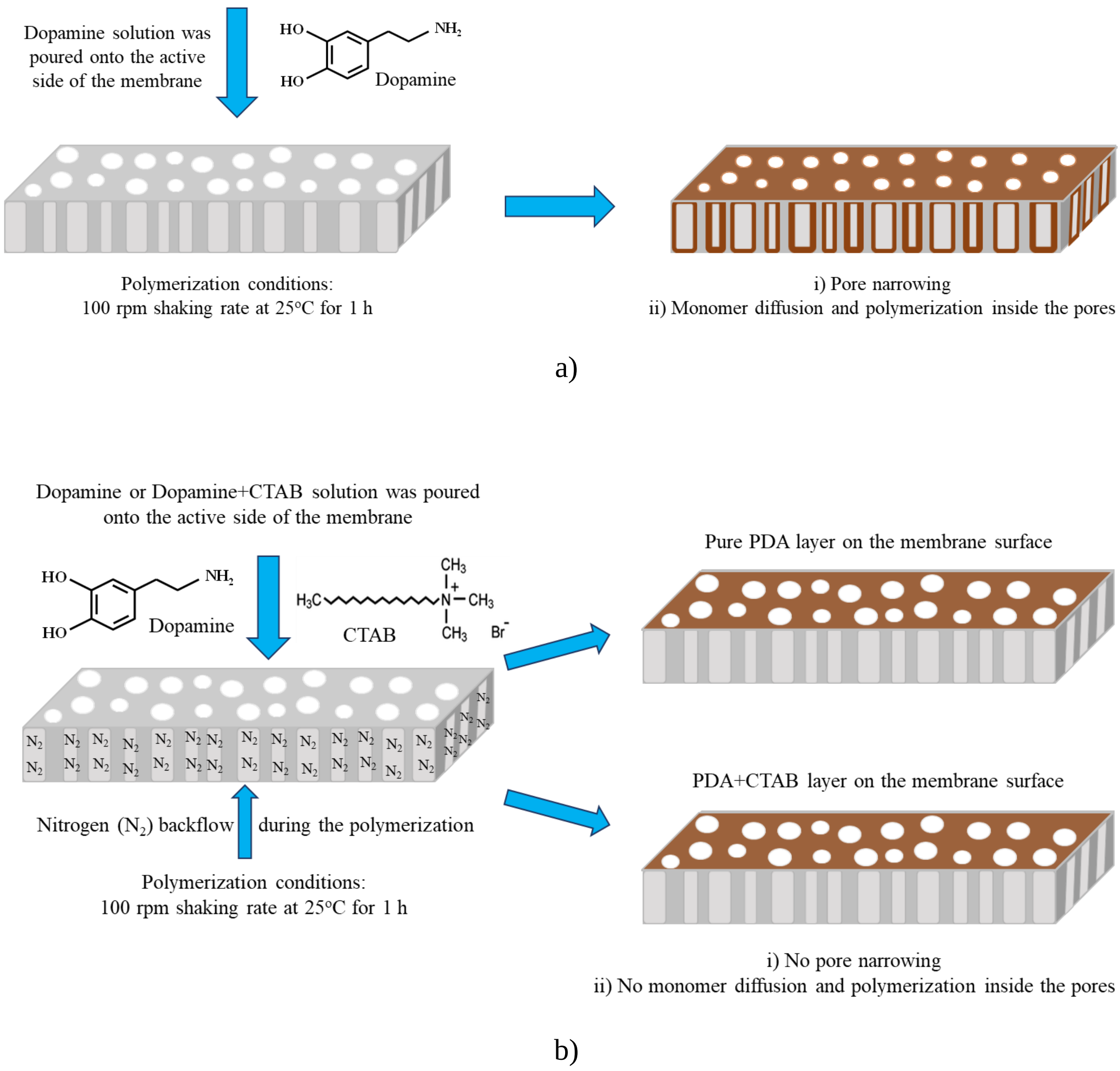


**Scheme S1.** Polymerization methods used in the study a) Conventional polymerization, b) PolyPrev polymerization

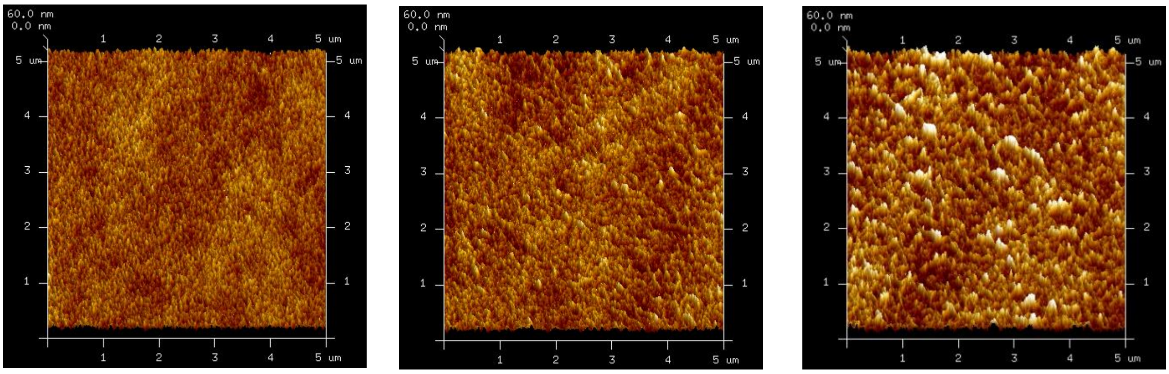


PES$_{\text{Support}}$ PES_PDA$_{\text{Conv Poly}}$ PES_PDA$_{\text{PolyPrev Poly}}$

**Figure S1.** AFM images of the support and PDA coated membranes.

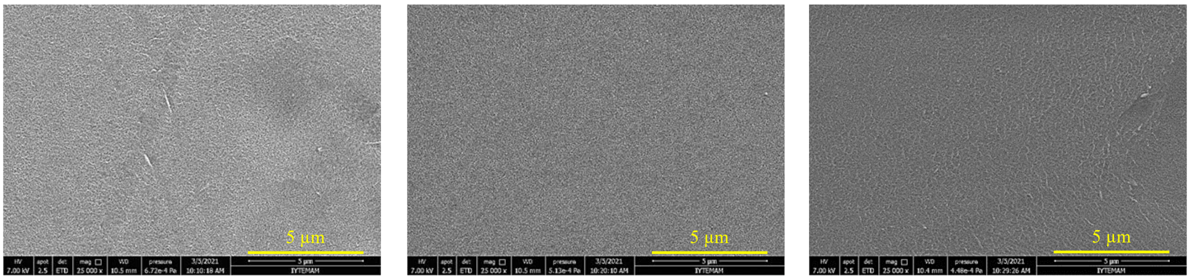


PES_PDA+CTAB$_{\text{LCMC}}$ PES_PDA+CTAB$_{\text{CMC}}$ PES_PDA+CTAB$_{\text{HCMC}}$

**Figure S2.** SEM images of active surface side of the co-deposited membranes (25kX magnification).

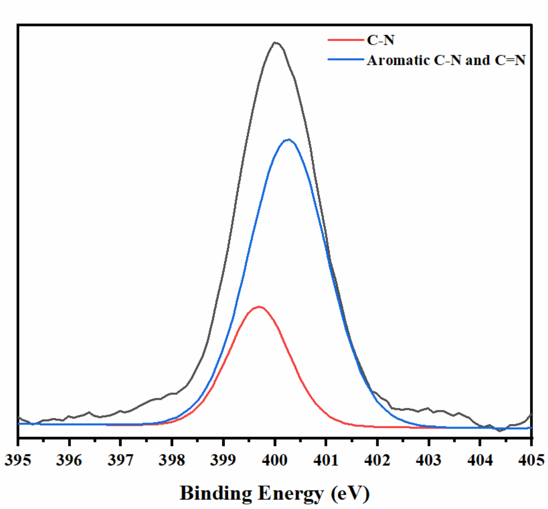


a) PES$_{\text{Support}}$

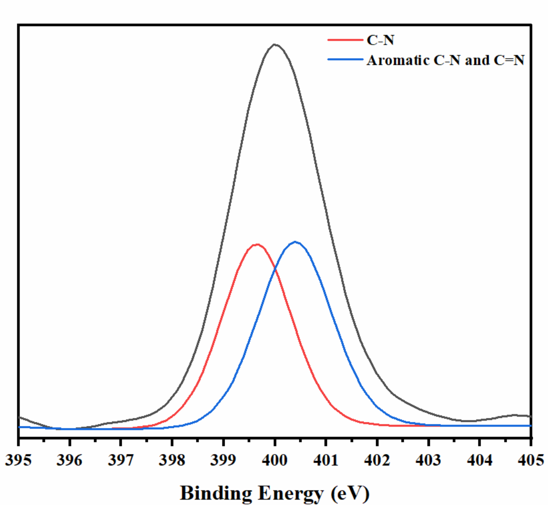


b) PES_PDA$_{\text{PolyPrev Poly}}$

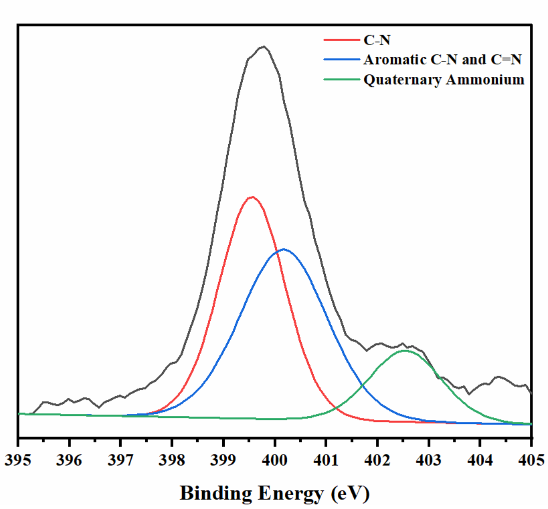


c) PES_PDA+CTAB$_{\text{LCMC}}$

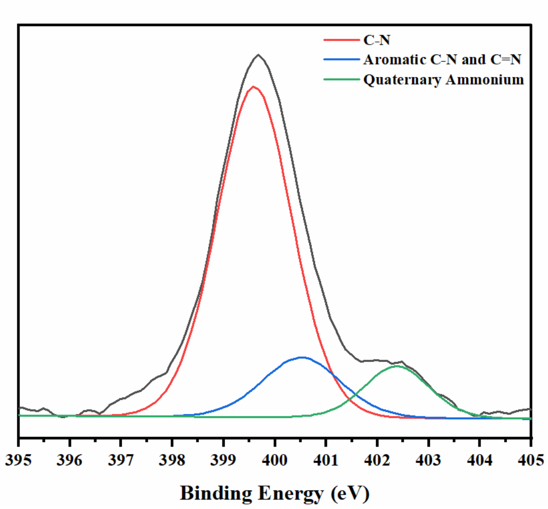


d) PES_PDA+CTAB$_{\text{CMC}}$

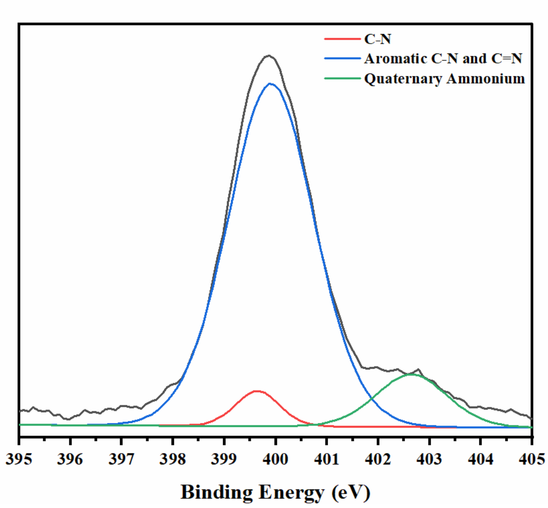


e) PES_PDA+CTAB$_{\text{HCMC}}$

**Figure S3.** High-resolution XPS spectra of the N1s region for the membranes.

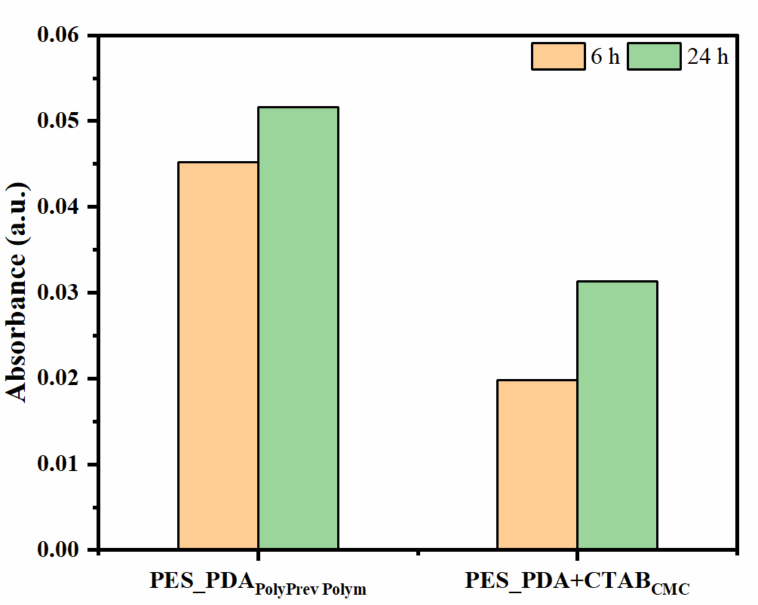


**Figure S4.** The absorbance of PDA released from the membrane surface after 6 and 24 h.

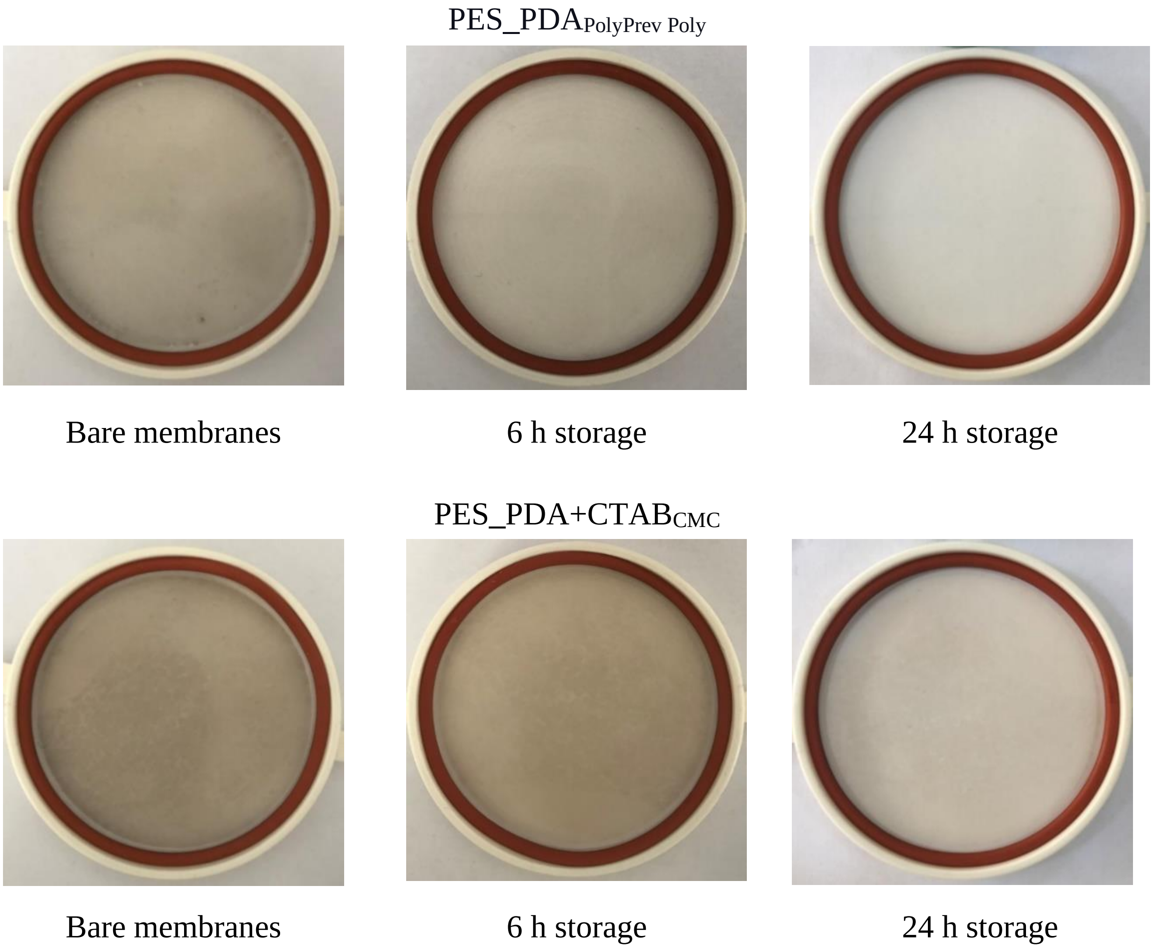


**Figure S5.** Digital images of the top active side of the membranes after alkaline stability.

**Table S1.** Surface properties of the support and PDA coated membranes.

| Membranes | $R_a$ (nm) | $R_q$ (nm) | Pore Diameter (nm) |
|---|---|---|---|
| $PES_{Support}$ | 4.36±0.03 | 5.54±0.05 | 27.8±4.3 |
| $PES\_PDA_{Conv\ Poly}$ | 4.90±0.09 | 6.17±0.09 | 18.9±1.8 |
| $PES\_PDA_{PolyPrev\ Poly}$ | 5.98±0.26 | 7.51±0.31 | 26.4±4.1 |

**Table S2.** Contact angle measurements of the support, PDA coated and co-deposited membranes.

| Membranes | Contact Angle ($^o$) |
|---|---|
| $PES_{Support}$ | 61.4±3.7 |
| $PES\_PDA_{Conv\ Poly}$ | 44.4±2.4 |
| $PES\_PDA_{PolyPrev\ Poly}$ | 46.7±1.9 |
| $PES\_PDA+CTAB_{LCMC}$ | 65.3±1.1 |
| $PES\_PDA+CTAB_{CMC}$ | 67.7±1.4 |
| $PES\_PDA+CTAB_{HCMC}$ | 63.9±1.8 |

**Table S3.** Membrane characteristics and performance after various surface modifications, as reported in the literature.

| Support | MWCO of Support (kDa) | Modification Method | Modification Agent | Modification Time (hours) | Flux Reduction (%) | Refs. |
|---|---|---|---|---|---|---|
| UF_PES | ~20 | Co-deposition | PDA+QPEI | 12 | 74 | 1 |
| UF_PES | 150 | UV-graft | Poly(CBOH) | ~9* | 57 | 2 |
| UF_PES | ~135 | UV-graft<br>Vacuum filtration | P, GO | 16* | 47 | 3 |
| UF_PSF | 20 nm diameter | UV-graft | MBHBA | ~0.5* | 36 | 4 |
| NF_HPAN | 50 | Co-deposition | PDA+rGOC | 24*/** | 80 | 5 |
| RO_PA | - | SI-ATRP | CAA+TMA | 2.5* | ~42 | 6 |
| RO_PA | - | SI-ATRP | pMEDSAH | ~2.5* | ~34 | 7 |
| RO_PA | - | Modification | PDA, MPC-co-AEMA | 27*** | ~10 | 8 |
| UF_PES | 150 | Co-deposition | PDA+CTAB | 1 | No reduction | This work |

* Three different steps were applied for modification. The time given in the table has included all processes.
** $CuSO_4$ (5 mM) and $H_2O_2$ (19.6 mM) were used as triggers for PDA polymerization.
*** Two different steps were applied for modification. The time given in the table has included all processes.

UF: Ultrafiltration, NF: Nanofiltration, RO: Reverse Osmosis, PES: Polyethersulfone, PSF: Polysulfone, PA: Polyamide, PDA: Polydopamine, QPEI: Quaternized polyethylenimine, Poly(CBOH): Poly(2-((2-hydroxy-3-(methacryloyloxy)propyl) dimethylammonio)acetate), P: Zwitterion polyampholyte hydrogel, GO: Graphene oxide nanosheets, MBHBA: N-(5-methyl-3-isobutyl-2-hydroxy-benzyl)-acrylamide, HPAN: Hydrolyzed Polyacrylonitrile, rGOC: Reduced Graphene Oxide-Copper, SI-ATRP: surface-initiated atom transfer radical polymerization, CAA: anionic 2-carboxyethyl acrylate, TMA: cationic [2-(acryloyloxy)ethyl] trimethyl ammonium chloride, pMEDSAH: poly[2-(methacryloyloxy)ethyl-dimethyl-(3-sulfopropyl) ammonium hydroxide], MPC-co-AEMA: 2-(methacryloyloxy) ethyl phosphorylcholine (MPC) copolymer with 2-aminoethyl methacrylate (AEMA), CTAB: Cetyltrimethylammonium bromide.

**References**

(1) Yao, L.; He, C.; Chen, S.; Zhao, W.; Xie, Y.; Sun, S.; Nie, S.; Zhao, C. Codeposition of Polydopamine and Zwitterionic Polymer on Membrane Surface with Enhanced Stability and Antibiofouling Property. *Langmuir* **2019,** *35*, 1430-1439.

(2) Weinman, S. T., Bass, M., Pandit, S., Herzberg, M., Freger, V., Husson, S. M. A Switchable Zwitterionic Membrane Surface Chemistry for Biofouling Control. *J. Membr. Sci.* **2018,** 5*48*, 490-501.

(3) Zhang, W.; Cheng, W.; Ziemann, E.; Bear, A.; Lu, X.; Elimelech, M.; Bernstein, R. Functionalization of Ultrafiltration Membrane with Polyampholyte Hydrogel and Graphene Oxide to Achieve Dual Antifouling and Antibacterial Properties. *J. Membr. Sci.* **2018***, 565*, 293-302.

(4) Xueli, G.; Haizeng, W.; Xing, H.; Congjie, G. Surface-Modified PSF UF Membrane by UV-Assisted Graft Polymerization of Capsaicin Derivative Moiety for Foulings and Bacterial Resistance. *J. Membr. Sci.* **2013,** *445,* 146-155.

(5) Zhu, J.; Wang, J.; Uliana, A. A.; Tian, M.; Zhang, Y.; Zhang, Y.; Volodin, A.; Simoens, K.; Yuan, S.; Li, J.; Lin, J.; Bernaerts, K.; Bruggen, B. V. Mussel-Inspired Architecture of High-Flux Loose Nanofiltration Membrane Functionalized with Antibacterial Reduced Graphene Oxide-Copper Nanocomposites. *ACS Appl. Mater. Interfaces* **2017,** *9, 34,* 28990-29001.

(6) Yang, Z.; Saeki, D.; Takagi, R.; Matsuyama, H. Improved Anti-biofouling Performance of Polyamide Reverse Osmosis Membranes Modified with a Polyampholyte with Effective Carboxyl Anion and Quaternary Ammonium Cation Ratio. *J. Membr. Sci.* **2020,** *595,* 117529.

(7) Yang, Z.; Zhang, X.; Xie, M.; Wu, H. C.; Yoshioka, T.; Saeki, D.; Matsuyama, H. Antifouling Thin-Film Composite Membranes with Multi-defense Properties by Controllably Constructing Amphiphilic Diblock Copolymer Brush Layer. *J. Membr. Sci.* **2020,** *614,* 118515.

(8) Karkhanechi, H.; Takagi, R.; Matsuyama, H. Enhanced Antibiofouling of RO Membranes via Polydopamine Coating and Polyzwitterion Immobilization. *Desalination* **2014,** *337*, 23-30.
